# Professional learning supports teacher well-being through needs-supplies fit and control-value processes

**DOI:** 10.1038/s41598-026-57946-9

**Published:** 2026-08-20

**Authors:** Fernando Núñez-Regueiro, Helen M. G. Watt, Paul W. Richardson, Herbert W. Marsh, Reinhard Pekrun

**Affiliations:** 1 LaRAC, Université Grenoble Alpes, 38000 Grenoble, France; 2 School of Education and Social Work, Faculty of Arts and Social Sciences, The University of Sydney, Sydney, NSW 2006 Australia; 3 Faculty of Education, Monash University, 19 Ancora Imparo Way, Melbourne, VIC 3800 Australia; 4 Institute for Positive Psychology and Education, Australian Catholic University, North Sydney, 2060 Australia; 5 University of Geneva, Geneva, Switzerland; 6 Oxford University, Oxford, UK; 7 University of Essex, Colchester, UK

**Keywords:** Teacher well-being, Continuous professional learning, Needs-supplies fit, Control-value theory, Cubic response surface analysis, Health care, Psychology, Psychology

## Abstract

Teacher well-being has become a critical challenge for contemporary education systems, yet the role of continuous professional learning (CPL) in supporting it remains poorly understood. We advance an integrative framework combining needs-supplies fit theory and control-value theory to explain when CPL supports teachers’ self-efficacy and, in turn, their well-being. Using nationally representative TALIS 2024 data from secondary teachers in France (*N* = 2877) and Australia (*N* = 3035), we show that CPL effects on teacher self-efficacy depend on needs-supplies fit: Supplies more strongly relate to self-efficacy when they exceed needs for CPL, whereas deficient supplies relate negatively to self-efficacy. Moreover, well-being levels peak when teachers both feel self-efficacious and value their work, but drop when either appraisal is lacking. These findings delineate conditions under which CPL may meaningfully support teacher well-being, with implications for systems confronting rising teacher attrition rates.

**Supplementary Information:**

The online version contains supplementary material (SI Text, SI Statistics) available at 10.1038/s41598-026-57946-9.

## Introduction

The teacher attrition problem is a pressing issue of today’s educational systems worldwide, with 6.5% of qualified teachers leaving the profession every year (OECD average in 2022–2023), corresponding to nearly 30% over a five-year period^1^. This creates challenges in filling teaching positions and may reduce teaching quality due to an increased reliance on unqualified teachers, who account for approximately 8% of the workforce on average^1^. Improving teacher well-being is key to addressing this problem, but multiple factors are at play. The first is the “reality shock” that teachers experience, notably when their strong vocational calling to work with youth and contribute to society^2^ is confronted with the difficulties of classroom stressors such as student misbehavior^3,4^. This can erode teachers’ confidence and enthusiasm, leading to early exit from the profession. A second factor concerns the quality of teacher education programs, which many preservice teachers view as insufficiently connected to practice and as eroding their motivation to graduate or pursue a career due to the accumulation of conflicting demands (e.g., academic dissertations, exams, internships, competitive entry examinations), thereby diminishing entries into the profession^5–7^. A third, more structural factor concerns the perceived decline in working conditions and professional status, particularly since the early 2000s in Western and economically-developed countries^2,8,9^. Accumulating teaching and administrative tasks, accountability pressures, and stagnant salaries (relative to other professions) have contributed to a sense that teaching is both demanding and undervalued^10,11^.

Together, these factors can contribute to creating difficult teaching conditions that undermine teachers’ well-being. Consistent with multidimensional frameworks of teacher well-being^11,12^, we use “teacher well-being” as an umbrella term for teachers’ positive functioning at work and, more specifically, the extent to which they enjoy teaching, are satisfied with their job and envisage staying in the profession. These indicators reflect complementary and interrelated dimensions of positive functioning, namely affective engagement with teaching practice (enjoyment), cognitive appraisal of one’s professional situation (job satisfaction), and behavioral commitment to the profession (intention to stay). When enjoyment fades and dissatisfaction becomes salient, many decide to quit. For instance, attrition rates over a 5-year period in countries such as France or Australia amount to 10%-15% of the teaching workforce^1,13^, while self-reported rates of intentions to leave within 5 years rates are even higher, reaching 18% in France and 28% in Australia^14^. These figures suggest that many strained teachers remain in the profession due to economic or other reasons, with potential negative consequences for student motivation and learning^15^.

Several strategies have been proposed to enhance teacher well-being. These include mentoring programs to reduce early stress and foster self-efficacy among teacher novices^16^; teacher education changes to strengthen links between training and teaching practices^3,6^; as well as policy reforms to lessen bureaucratic organization of schools^17^ and to increase teacher salaries, public recognition, and working conditions^2,10,11^. According to the Teaching and Learning International Survey (TALIS), a central strategy to improve teaching quality is continuous professional learning (CPL), understood as teachers’ participation in learning activities that help them update or extend their professional skills throughout their career. CPL includes formal training opportunities such as courses, education conferences, or formal qualification programs; but also informal training via visits to other schools, observation of others’ practices, or reflective analysis^18^. Viewed as a dynamic, career-long process of professional growth, CPL may represent a critical lever to foster teacher well-being and retention. While promising, CPL has been mainly considered in relation to its effects on students’ learning^18,19^. Most CPL research—and teachers themselves—continue to view professional learning as skill acquisition rather than as a process shaping teachers’ subjective experience and as a potentially powerful resource for renewal, rejuvenation or ‘remoralisation’ at a time of professional crisis^20^. As a result, the extent to which CPL may play a protective role for teachers themselves remains insufficiently understood and also undertheorized. To what extent, and through which mechanisms, might CPL promote teacher well-being?

**Fig. 1:**
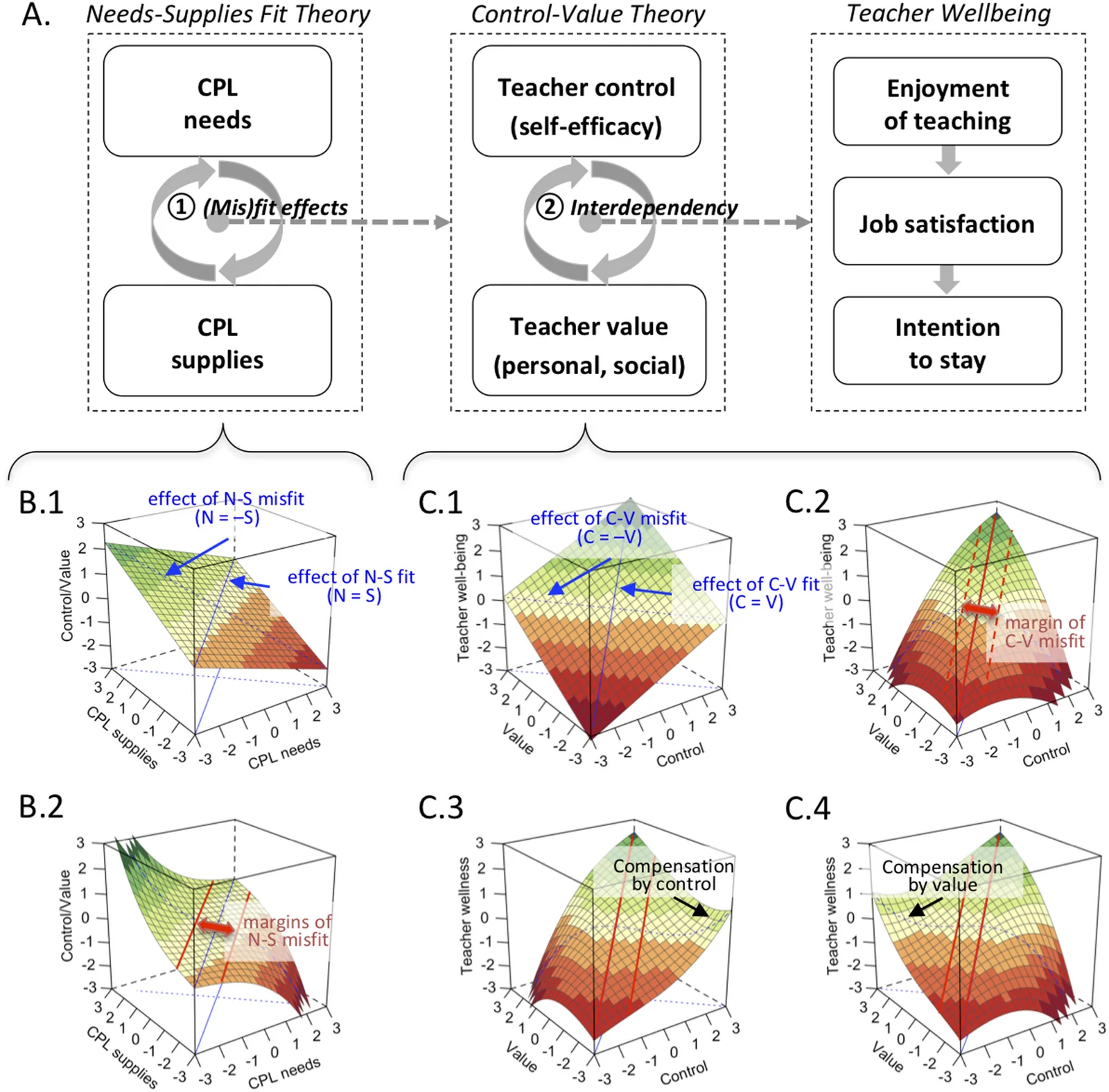
Teacher Well-Being Framework Integrating Needs-Supplies Fit and Control-Value Theories. CPL = Continuous professional learning. Needs and supplies in CPL are posited as predictors of teacher control (self-efficacy), augmenting control when supplies exceed needs, and decreasing it when supplies are deficient^21,23,29^. In turn, high teacher control fosters teacher well-being, notably when it coincides with high teacher value (personal utility, social utility)^26,30,31^. This integrative framework **(A)** thus provides a psychological account of the mechanisms linking teachers’ learning activities (CPL) and teacher well-being. Alternative hypotheses for needs-supplies fit effects **(B.1**,** B.2)** and control-value effects **(C.1-C.4)** guide the response surface analyses of TALIS datasets. Red lines correspond to misfit effects reversing (reversal) or becoming sizable (acceleration). Variable values reflect standardized levels (z-scores) in the population: < 0 = low; 0 = average; >0 = high.

The present framework integrates needs-supplies (N-S) fit theory^21–23^ and control-value (C-V) theory^24–26^ to explain how teachers’ CPL shapes their enjoyment, satisfaction and intention to stay in the teaching profession (Fig. 1A). The integration lies in proposing that these two processes are embedded in teacher well-being. Specifically, the degree of match between needs and supplies in CPL is assumed to be related to the degree to which teachers feel in control; in turn, increases in control may not suffice to promote well-being if teachers do not, at the same time, attach value to their profession. We examine this proposition by first comparing alternative forms (linear and nonlinear) of N-S fit effects on C-V constructs, and then alternative forms of C-V effects on well-being indicators. Through this sequential analytic strategy that accounts for the complexity of N-S fit and C-V effects, we aim to examine the coherence of this integrated teacher well-being framework, thereby following calls for theoretical integration and precise testing in motivation research^27,28^. Using TALIS data, we find that CPL supplies positively relate to teachers’ self-efficacy only when exceeding their needs, and teacher well-being is highest when control and value co-occur. These patterns are consistent across France and Australia samples. Together, they support the proposed integrative framework.

### From teachers’ needs-supplies fit in CPL to their sense of control and value

N-S fit represents one of three major forms of person-environment fit, alongside demands-resources fit and supplementary fit^21,22,32,33^. Whereas the latter focus on the interplay between task demands and task resources^34,35^, and between organizational and individual characteristics (e.g., goals, values, skills)^33,36^, N-S fit focuses on the balance between contextual supplies in a domain (e.g., perceived autonomy at work) and individual needs for such supplies (e.g., preferred autonomy at work)^21,23,29^. This approach is operationalized through measures asking individuals to rate their perceived requirements (“needs”) versus actual levels (“supplies”) in a given resource. In TALIS, needs and supplies are measured in relation to professional learning activities in three domains: What to teach, how to teach, and to whom (see Table 1). These domains are highly interrelated (*r* = .73 to 0.88) and encompass global teaching skills^37^.

**Table 1:** Teachers’ Continuous Professional Learning Needs and Supplies in TALIS 2024.

Item stem for CPL needs	Item stem for CPL supplies	Exemplary CPL Items
Needs: For each of the areas listed [CPL items], please indicate the extent to which you currently need professional learning activities. Response scale:4 = no need at present; 1 = high level of need (reverse-coded)	Supplies: Were any of the topics listed [CPL items] included in your professional learning activities during the last 12 months? Response scale: 1 = Yes; 2 = No (reverse-coded).	*What to teach*: . Knowledge and understanding of my subject field(s) . The pedagogy of the subject matter(s) I teach . Knowledge of the curriculum *How to teach*: . Analysis and use of student assessments . Classroom management for student behavior . Teacher-parent or guardian co-operation *Whom to teach*: . Teaching students with special education needs . Teaching in a multicultural or multilingual setting . Approaches to individualized learning

Note. Fifteen CPL items are assessed. An additional item, “Knowledge and understanding of environmental sustainability”, reflects OECD policy priorities on sustainability education rather than a core component of teachers’ CPL, and was therefore excluded from the present analyses. Needs and supplies levels are matched through standardization of metrics (z-scores; see Method).

Using polynomial regression and response surface analysis^38–40,60^, it is possible to assess how the degree of fit or misfit between needs and supplies in CPL relates to teacher outcomes. Prior findings in organizational and educational contexts found that optimal outcomes are achieved when high amounts of supplies exceed (positive misfit) levels of needs, whereas deficient supplies relate to diminished feelings of competence, disengagement and dissatisfaction^21,23,29,33^. On this basis, it is expected that teacher self-efficacy—tapping control—will be highest when CPL supplies exceed teachers’ levels of CPL needs. Two N-S fit patterns can be derived: One linear, positing that the intensity of misfit does not change the structural relations at play (Fig. 1, B.1); and one non-linear (cubic), positing that the effect of positive and negative N-S misfit increases as the degree of misfit becomes larger (Fig. 1, B.2), a more realistic prediction according to prior research^23,29^. Similar predictions can be made for teacher value, but current evidence suggests such link is likely weak. Teaching motivations, which include personal and social values, are indeed anchored in processes of initial career determination^2,41^ and may not be influenced directly by evolving needs in CPL.

### From teacher control-value to teacher well-being

Control-value theory^24,25,26^ provides the cognitive-emotional complement to link N-S fit processes to teacher well-being (Fig. 1). Specifically, C-V theory explains how teachers’ perceptions of control and value influence the quality of emotions, satisfaction, and intentions to remain in the profession^30^.

In this framework, “control” refers to teachers’ perceived capability to influence classroom processes and student outcomes (see also the related construct of “expectancy” in situated expectancy-value theory^42^). In TALIS, this is operationalized through measuring self-efficacy beliefs to manage the classroom, to engage students in learning, and to use diverse instructional approaches to support students’ learning needs^18,43^. By contrast, “value” reflects the perceived importance and meaningfulness of teaching, operationalized in TALIS by measuring personal utility values (e.g., job security) and social utility values (e.g., make a social contribution)^2,18,41^.

In our integrative framework (Fig. 1), N-S fit and C-V theories are related through teacher control (i.e., self-efficacy): When professional learning opportunities (CPL supplies) adequately match teachers’ learning development (CPL needs), feelings of control over teaching processes are reinforced. However, according to C-V theory, control alone is insufficient to promote positive teacher emotions if it is not accompanied by value^30^. This is because teachers who feel in control but attach little value to their work may experience negative emotions such as boredom or sadness. Conversely, teacher who greatly value their work, but feel little confidence in teaching well, will likely experience negative emotions such as anxiety or anger^30,44^. In other words, high control combined with high value is necessary to yield optimal levels of positive emotions such as teacher enjoyment. In the same way, optimal levels of job satisfaction and intention to stay require both control and value to occur.

Mathematically, combinatory effects between two predictors can be characterized in different ways representing independent, interdependent, or compensatory mechanisms^45,36^. In the independent hypothesis (Fig. 1, C.1), control and value may have additive, linear effects on teacher well-being (i.e., enjoyment, job satisfaction, intention to stay), with peak well-being occurring when both are present^3,12,46^. In this case, the effects are independent in the sense that levels on one construct (e.g., low control) will not modify the effect of the other (e.g., high value). By contrast, control and value may be functionally interdependent in their effects (Fig. 1, C.2), meaning that the effect of control on well-being is a function of value levels (and conversely), with effects diminishing in intensity with decreasing levels in the other construct. Several studies have reported evidence for this functional interdependence on student outcomes^47–49^. In Response Surface Analysis (RSA) terms, this is represented by a congruence effect, in which well-being levels increase as control and value levels grow together, but drop as soon as either teacher control, or value, become low (i.e., z-score < 0; Fig. 1, C.2). This translates into a positive trend over the line of fit (where * control=value*) and a concave or inverted-U-shaped curvature over the line of misfit (where * control=-value*). Finally, alternative hypotheses modify this functional interdependence through compensatory mechanisms, in which high control may compensate for low value (Fig. 1, C.3), or conversely (Fig. 1, C.4)^48^, in the form of cubic or S-shaped curvature over the line of C-V misfit. Such compensatory mechanisms would sustain teacher well-being at intermediate levels despite discrepant control-value levels, although optimal levels of well-being would still occur when both control and value are high.

## Present study

Building on the integration of N-S fit and C-V theories, the present study aimed to analyze how teachers’ CPL experiences are associated with their sense of control and value, and how these, in turn, relate to teacher well-being indicators (i.e., enjoyment, job satisfaction, intention to stay). To this end, we mobilized the powerful TALIS 2024 datasets, in the French and Australian contexts. France and Australia were selected because they are comparable in terms of human development, face significant teacher attrition, share broadly similar motivational profiles among teachers^6,41^, but differ structurally in CPL provision (see Results)^50^, enabling a robust cross-system test of the model. We also focused on secondary education because teacher attrition rates and related issues (i.e., unfilled teaching vacancies, overreliance on nonqualified teachers) are consistently higher than in primary education^1,17,52^, making this level particularly relevant for testing our theoretical model. Consistent with the integrated framework (Fig. 1), the analyses proceeded sequentially, first examining how CPL experiences (N-S fit) related to teacher control and value, and then how control and value related to teacher well-being indicators. Two research questions guided the analyses:

1) Do CPL supplies relate significantly to teachers’ control and value processes, and does this depend on their CPL need levels?

2) Are there effects of control and value on teacher well-being, and are these effects functionally independent or interdependent?

Based on N-S fit theory and prior research, we expected self-efficacy (control) to be highest when CPL supplies exceeded CPL needs, with misfit effects (positive or negative mismatches) intensifying with increasing degrees of misfit (Fig. 1, B.2)^23,29^. We did not expect N-S fit to relate to teachers’ value appraisals (social or personal utility values)^31^. Based on C-V theory^26,30^, we expected that control and value would jointly predict teacher well-being, producing higher well-being when both were high and lower well-being when either was low, possibly through diverse patterns reflecting additive effects, interdependence, or compensatory mechanisms (Fig. 1, C.1-C.4). To address these questions, we used response surface analysis, an optimal technique to detect complex interactive processes in psychological functioning^29,35,39,45,51^.

## Results

### Descriptive statistics

Table 2 provides descriptive statistics for all study variables in France and Australia. For N-S fit constructs, teachers reported moderate CPL needs in both contexts (M = 2.3 on a 1–4 scale), but higher CPL supplies in France than Australia (M = 0.7 vs. 0.4, on a 0–1 metric), indicating that French teachers had a higher probability of having recently benefited from learning activities in the themes of professional development assessed (see items in Table 1). These qualitative differences in thematic coverage appeared linked to quantitative differences in access to CPL formats (see Fig. S1, *SI Text*): In France, participation rates were strong across 8 major formats (60–90%), including qualification programs (90%), mentoring (79%), and teacher networks (75%), whereas participation was more modest in Australia (26–88% overall, 88% qualification programs, 67% mentoring, 26% teacher networks).

**Table 2:** Descriptive Statistics.

	M	SD	1.	2.	3.	4.	5.	6.	7.	8.
1. CPL needs	2.3/2.3	0.6/0.5	—	− 0.030	− 0.240***	0.068	− 0.003	− 0.123**	− 0.063	− 0.021
2. CPL supplies	0.7/0.4	0.2/0.2	0.051*	—	0.221***	0.037	0.144***	0.114***	0.207***	0.072†
3. Self-efficacy	3.1/3.2	0.5/0.5	− 0.244***	0.102***	—	0.070†	0.189***	0.345***	0.219***	0.057
4. Perso. utility	3.5/3.5	0.4/0.4	− 0.019	0.072**	0.046†	—	0.200***	− 0.041	0.030	0.036
5. Social utility	3.5/3.4	0.6/0.6	0.016	0.042†	0.190***	0.089***	—	0.267***	0.203***	0.158***
6. Enjoyment	3.4/3.4	0.5/0.5	− 0.126***	0.104***	0.364***	− 0.019	0.270***	—	0.494***	0.239***
7. Job satisf.	2.8/2.7	0.5/0.2	− 0.057*	0.112***	0.293***	0.033	0.199***	0.580***	—	0.385***
8. Inten. to stay	0.5/0.5	0.2/0.3	− 0.067†	0.080**	0.111***	0.040	0.123***	0.268***	0.307***	—

Note. *N* = 2877 (France); 3035 (Australia) secondary school teachers. Values for France appear first (M, SD) and below the diagonal (correlations); values for Australia appear second and above the diagonal.

†*p* < .10; *p < .05; **p < .01; ***p < .001.

Concerning C-V constructs, teachers’ average self-efficacy and personal and social utility values were high in both contexts (M = 3.1–3.5 on 1–4 scale). Correlations between control and value constructs were modest (*r* = .05-.20), consistent with the idea that feeling capable does not entail finding meaning in teaching^30^. Furthermore, personal and social utility values correlated more weakly in France (*r* = .09, *p* < .001) than in Australia (*r* = .20, *p* < .001), suggesting that opportunities for experiencing both job-related benefits and social contribution motives may be more limited in the French context^2,41^.

The teacher well-being indicators formed a coherent cluster, with strong positive correlations between teaching enjoyment and job satisfaction (*r* = .49–.58, *p* < .001) and moderate correlations with intention (*r* = .24-.39, *p* < .001). Consistent with C-V theory, self-efficacy and social utility value were moderately to strongly related with teacher well-being (*r* = .20-.36 with enjoyment and job satisfaction, *p* < .001). By contrast, personal utility value was not correlated with it (*r* =-.04-.04, *p* > .10). Such patterns suggest that only certain kinds of teacher value (i.e., social utility, not personal utility) may matter for teacher well-being. CPL needs and supplies also correlated weakly with teacher well-being (around *r* = − .02 to − .13 for needs, *r* = .08-.21 for supplies), which could reflect the interplay of their effects (N-S fit or misfit processes), but also unequal mediation processes across C-V constructs (interdependence effects) (see Fig. 1). The following sections shed light on these preliminary insights by disentangling the specific effects of N-S fit and C-V constructs via polynomial regression modeling.

### Needs-supplies fit effects on control-value constructs (RQ1)

For each C-V construct (i.e., self-efficacy, personal utility value, social utility value), we compared 37 polynomial families of varying degrees of complexity, accounting for linear, quadratic and cubic patterns of N-S fit effects (for full comparisons, see *SI Statistics*). Best-fitting solutions were identified based on parsimony indices (AIC and BIC weights), explained variance (R^2^ adjusted for complexity), fit indices (CFI, RMSEA, SRMR), and significant higher-order effects (Wald tests). In both French and Australian samples, final models showed excellent fit to the data (Table 3). For value constructs, the effects of CPL needs were not significant ($\:{b}_{1}$, * p>.05*), whereas the effects of CPL supplies were significant but small ($\:{b}_{2}$, * p<.05*); one interaction effect also occurred ($\:{b}_{4}=-0.106,\:p=.005$) (Table 4). However, in each case, the total explained variance was trivial in size (R^2^_adj_ ≤ 2%), meaning that N-S effects on value constructs were not meaningful. This was reflected in response surfaces being mostly flat (see Fig. S3, *SI Text*), confirming that teacher value did not vary by CPL needs or supplies levels.

**Table 3:** Fit indices of models of needs-supplies fit and control-value effects.

Polynomial Model	$\:{\chi\:}^{2}$	df	*p*	wAIC	*R* ^2^_adj_	CFI	RMSEA	SRMR
CPL Needs-Supplies Effects on Teacher Control-Value								
France:								
Self-efficacy: cubic model (FM28)	0.7	3	0.862	0.323	0.079	1.000	0.000	0.001
Personal utility: linear model (FM2)	5.4	8	0.715	0.162	0.005	1.000	0.000	0.011
Social utility: quadratic model (FM4)	4.6	6	0.596	0.188	0.013	1.000	0.000	0.006
Australia:								
Self-efficacy: quadratic model (FM5)	8.6	6	0.196	0.063	0.123	0.991	0.012	0.006
Personal utility: quadratic model (FM6)	3.6	6	0.725	0.219	0.009	1.000	0.000	0.006
Social utility: linear model (FM2)	8.1	8	0.428	0.154	0.021	0.998	0.002	0.008
Control-Value Effects (Self-Efficacy*Personal Utility) on Teacher Well-Being								
France:								
Enjoyment: cubic model (FM19)	1.4	3	0.709	0.190	0.140	1.000	0.000	0.002
Job satisfaction: cubic model (FM24)	2.2	3	0.535	0.483	0.098	1.000	0.000	0.002
Intention to stay: quadratic model (FM5)	3.7	6	0.715	0.186	0.018	1.000	0.000	0.006
Australia:								
Enjoyment: cubic model (FM23)	0.4	3	0.945	0.380	0.133	1.000	0.000	0.001
Job satisfaction: cubic model (FM17)	5.9	3	0.119	0.075	0.052	0.979	0.019	0.004
Intention to stay : cubic model (FM17)	1.4	3	0.713	0.137	0.010	1.000	0.000	0.003
Control-Value Effects (Self-Efficacy*Social Utility) on Teacher Well-Being								
France:								
Enjoyment: cubic model (FM19)	1.3	3	0.720	0.234	0.187	1.000	0.000	0.002
Job satisfaction: cubic model (FM16)	1.9	3	0.593	0.226	0.134	1.000	0.000	0.002
Intention to stay: quadratic model (FM5)	4.9	6	0.563	0.121	0.027	1.000	0.000	0.007
Australia:								
Enjoyment: quadratic model (FM9)	9.2	6	0.161	0.051	0.164	0.989	0.014	0.005
Job satisfaction: linear model (FM3)	5.9	7	0.557	0.164	0.075	1.000	0.000	0.004
Intention to stay: linear model (FM5)	5.1	6	0.532	0.131	0.027	1.000	0.000	0.004

Note. Numbered models (e.g., “FM28”) correspond to the best-fitting polynomial family identified after comparison across 37 linear, quadratic, or cubic families (see *SI Statistics*). Model parameters and nontrivial response surfaces (R^2^_adj_ > 0.02) are reported in Tables 4, 5 and 6; Figs. 2, 3 and 4.

**Table 4:** Effects of CPL Needs and Supplies on Teacher Control-Value.

Variable	France			Australia		
	Self-efficacy (control)	Personal utility (value)	Social utility (value)	Self-efficacy (control)	Personal utility (value)	Social utility (value)
*Parameters*						
Intercept ($\:{b}_{0})$	-0.058†	0.000	0.005	-0.105***	-0.063†	0.000
Needs ($\:{b}_{1})$	-0.194***	0.000	-0.002	-0.241***	0.067†	0.000
Supplies ($\:{b}_{2}$)	0.032	0.073**	0.047†	0.220***	0.074**	0.146***
Needs^2 ($\:{b}_{3}$)	0.027	0.000	0.000	0.102***	0.000	0.000
Needs*Supplies ($\:{b}_{4}$)	0.005	0.000	-0.106**	0.000	0.000	0.000
Supplies^2 ($\:{b}_{5}$)	0.032	0.000	0.000	0.000	0.065**	0.000
Needs^3 ($\:{b}_{6}$)	-0.021***	0.000	0.000	0.000	0.000	0.000
Needs^2*Supplies ($\:{b}_{7}$)	0.062***	0.000	0.000	0.000	0.000	0.000
Needs*Supplies^2 ($\:{b}_{8}$)	0.000	0.000	0.000	0.000	0.000	0.000
Supplies^3 ($\:{b}_{9}$)	0.000	0.000	0.000	0.000	0.000	0.000
*Metaparameters*						
$\:{u}_{1}={b}_{1}+{b}_{2}$	-0.163***	0.073**	0.045	-0.021	0.140**	0.146***
$\:{u}_{2}={b}_{3}+{b}_{4}+{b}_{5}$	0.063	0.000	-0.106**	0.102***	0.065**	0.000
$\:{u}_{3}={b}_{6}+{b}_{7}+{b}_{8}+{b}_{9}$	0.041***	0.000	0.000	0.000	0.000	0.000
$\:{v}_{1}={b}_{1}-{b}_{2}$	-0.226***	-0.073**	-0.049	-0.461***	-0.007	-0.146***
$\:{v}_{2}={b}_{3}-{b}_{4}+{b}_{5}$	0.054	0.000	0.106**	0.102***	0.065**	0.000
$\:{v}_{3}={b}_{6}-{b}_{7}+{b}_{8}-{b}_{9}$	-0.082***	0.000	0.000	0.000	0.000	0.000
Explained var. (R2 adjusted)	0.079	0.005	0.013	0.123	0.009	0.021

Note. Metaparameters$\:{u}_{1}$to$\:{v}_{3}$correspond to the linear, quadratic and cubic N-S fit ($\:{u}_{1}$to$\:{u}_{3}$) or misfit ($\:{v}_{1}$to$\:{v}_{3}$) effects. Zero values (0.000) are fixed by construction, as part of the identification strategy of the best-fitting polynomial model. Fit indices for each model are provided in Table 3.

†*p* < .10; *p < .05; **p < .01; ***p < .001.

**Fig. 2:**
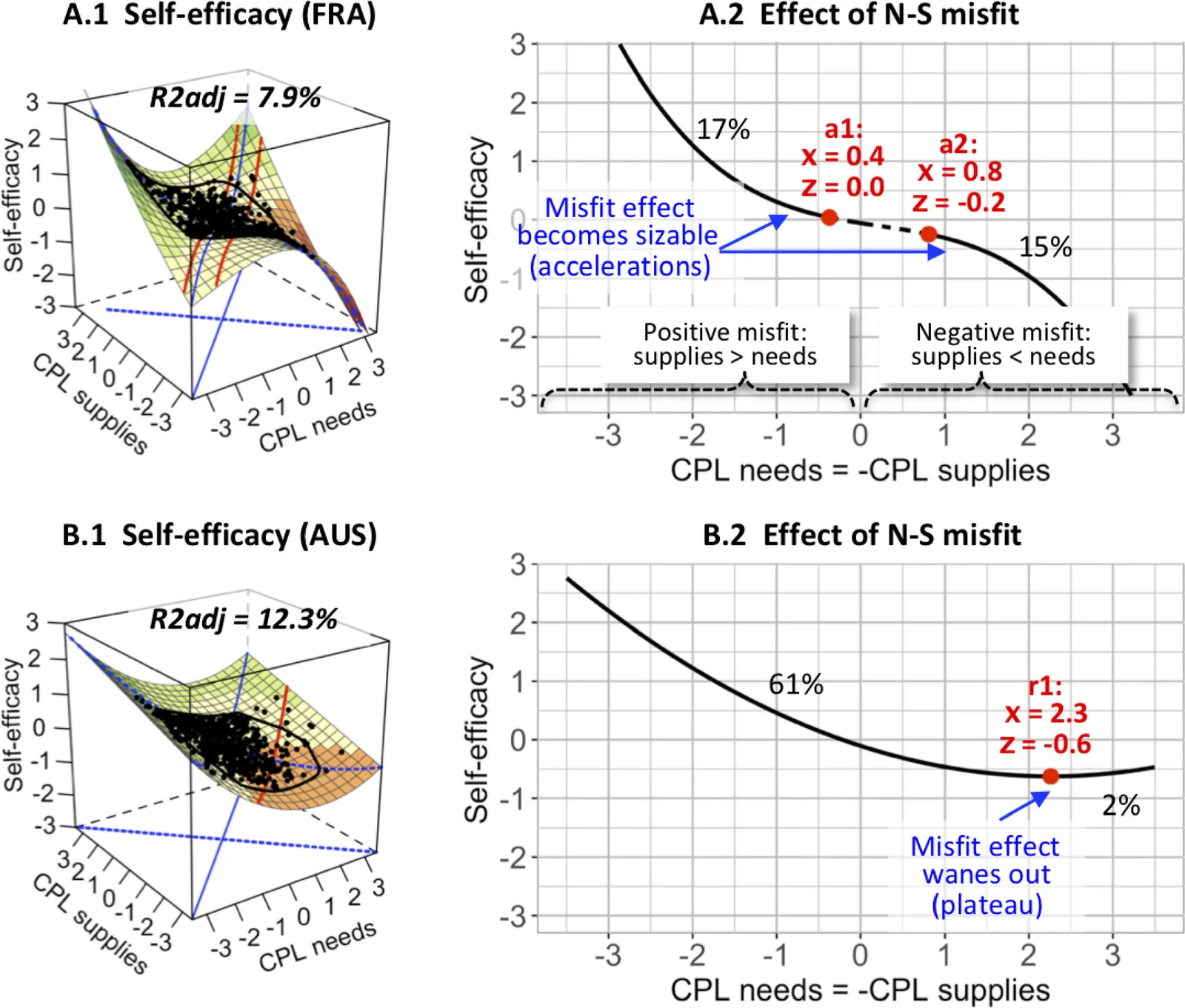
CPL needs-supplies fit effects on teacher self-efficacy. CPL = Continuous professional learning; FRA= France; AUS = Australia. In both contexts, the effects of supplies in professional learning (CPL supplies) depend on individual differences in professional learning requirements (CPL needs). Highest self-efficacy levels are reached when supplies exceed needs (positive misfit). The responses over the line of needs-supplies misfit show acceleration or reversals characteristics of nonlinearities (“a1”, “a2” and “r1” in panels A.2 and B.2), delimiting areas where misfit effects wane out or become sizable. Fit indices and model parameters are reported in Tables 3 and 4.

On the contrary, N-S fit effects on self-efficacy were nontrivial in size (R^2^_adj_ = 8% in France, 12% in Australia) and were characterized by significant quadratic or cubic nonlinearities (see metaparameters $\:{v}_{2}$ and $\:{v}_{3}$, *p* < .001, Table 4). In both contexts, highest self-efficacy levels were reached when supplies exceeded needs (positive misfit) and, secondly, when high supplies matched high needs (positive fit) (see Fig. 2). These effects were nonlinear, because they accelerated with increasing degrees of N-S misfit (France), or plateaued at high levels of misfit (Australia). More specifically, for France, the margins of acceleration were near average predictor values in the French sample (i.e., near 0: $\:{a}_{1}=0.4,{a}_{2}=0.8$, see red lines in Fig. 2, A.2), indicating that positive and negative N-S misfit effects occurred very quickly among French teachers, that is, as soon as needs and supplies differed in intensity. These accelerations were significant at * p<.05* for 32% of teachers (17% in positive misfit, 15% in negative misfit). In the Australian sample, the misfit effect increased in size with positive misfit, but gradually waned out and reversed with negative misfit, when supplies became largely deficient relative to needs ($\:{r}_{1}=2.3\:SD$, Fig. 2, B.2). The main trend of the misfit effect was nevertheless much more impactful (61% of teachers) than its reversal (2% of teachers). Besides differences in the pattern of nonlinearities, a common principle prevailed in both national contexts: CPL supplies effects on self-efficacy depended on individual differences in the level of CPL needs. This was also evidenced by the fact that these final models augmented the explained variance by 150% to 700%, relative to a model only including the effects of supplies.

### Control-value effects on teacher well-being (RQ2)

The same RSA methodology was applied to identify best-fitting models of C-V effects on teacher well-being indicators, based on fit to the data and parsimony indices. Two kinds of C-V combinations were evaluated, namely self-efficacy*personal utility and self-efficacy*social utility constructs. All final models showed again excellent fit (Table 3).

**Fig. 3:**
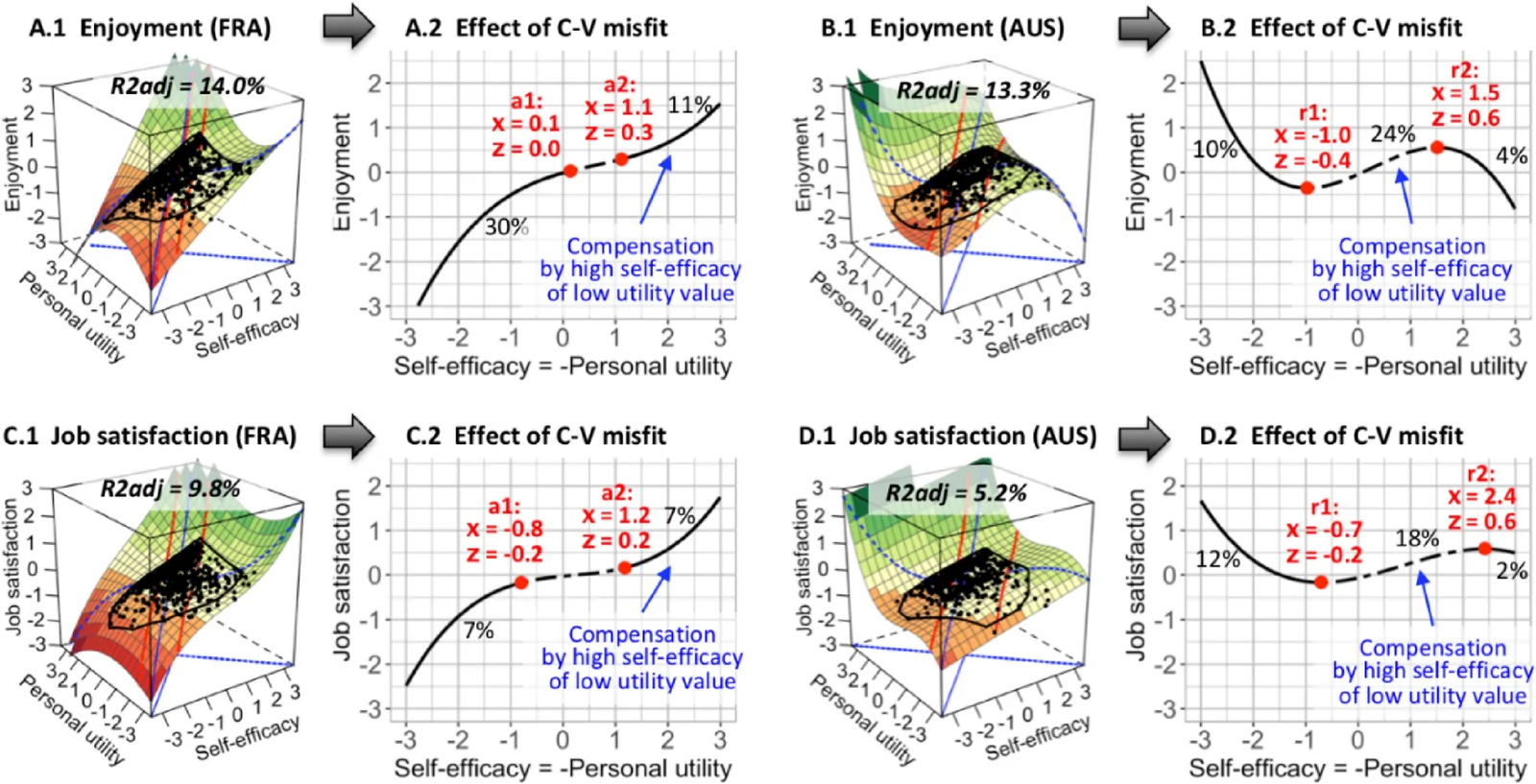
Control-value (self-efficacy*personal utility) effects on teacher well-being indicators. FRA= France; AUS = Australia. Most patterns show compensation by self-efficacy, whereby job satisfaction and enjoyment peak when self-efficacy and personal utility are above average (> 0), or when high self-efficacy (> 0) compensates for low personal utility (< 0). For 1/10 Australian teachers, high levels of personal utility (0.6–0.8 above the mean) may maintain teacher well-being, even if self-efficacy is lacking. Fit indices and model parameters are reported in Tables 3 and 5.

For the first combination (self-efficacy*personal utility), C-V effects on intention to stay were ignorable (R^2^_adj_ < 2%), but they accounted for 5% to 14% of the variance in teacher enjoyment and job satisfaction, in the form of cubic effects ($\:{v}_{3}$, *p* < .001, Table 5). The response surfaces aligned with a pattern of compensation by self-efficacy (Fig. 3): Highest well-being levels occurred when both personal utility value and self-efficacy were high; high self-efficacy maintained moderate levels of well-being in the absence of personal utility; but high personal utility could not compensate for low self-efficacy. This pattern significantly affected 14% to 41% of French teachers, and 18% to 24% of Australian teachers (see Fig. 3). For 2% to 4% of Australian teachers, the compensatory effect by self-efficacy reversed at extreme low levels of personal utility value (i.e., self-efficacy = -personal utility > 1.4 SD or > 2.4 SD, see $\:{r}_{2}$ thresholds, Fig. 3, B.2, D.2). Moreover, for 10% to 12% of Australian teachers, a marginal compensatory effect by personal utility occurred at low levels of self-efficacy (i.e., self-efficacy = -personal utility < -1.0 SD or < -0.7 SD, see $\:{r}_{1}$ thresholds, Fig. 3, B.2, D.2). No such reversals appeared in the French sample (Fig. 3, A.2, C.2).

**Table 5:** Effects of control (self-efficacy) and value (personal utility) on teacher well-being.

Variable	France			Australia		
	Enjoyment	Job satisfaction	Intention to stay	Enjoyment	Job satisfaction	Intention to stay
*Parameters*						
Intercept ($\:{b}_{0}$)	-0.013	-0.028	-0.001	-0.043	-0.067	-0.066
Self-efficacy ($\:{b}_{1})$	0.304***	0.168***	0.111***	0.447***	0.221***	0.053*
Personal utility ($\:{b}_{2}$)	-0.022	0.055†	0.036	-0.078*	-0.033	0.040
Self-efficacy^2 ($\:{b}_{3}$)	-0.013	0.003	0.000	-0.051*	-0.001	-0.040†
Self-efficacy*Personal utility ($\:{b}_{4}$)	0.109***	0.067*	-0.019	-0.033	-0.002	0.007
Personal utility ^2 ($\:{b}_{5}$)	0.013	0.027	0.000	0.115***	0.126*	0.156**
Self-efficacy^3 ($\:{b}_{6}$)	0.000	0.033***	0.000	-0.030**	0.000	0.000
Self-efficacy^2*Personal utility ($\:{b}_{7}$)	0.000	0.000	0.000	0.030**	0.000	0.000
Self-efficacy*Personal utility ^2 ($\:{b}_{8}$)	0.058**	0.033***	0.000	-0.030**	0.000	0.000
Personal utility ^3 ($\:{b}_{9}$)	0.000	0.000	0.000	0.030**	0.050*	0.044*
*Metaparameters*						
$\:{u}_{1}={b}_{1}+{b}_{2}$	0.282***	0.222***	0.147***	0.369***	0.188***	0.093*
$\:{u}_{2}={b}_{3}+{b}_{4}+{b}_{5}$	0.108**	0.097*	-0.019	0.030	0.124†	0.123†
$\:{u}_{3}={b}_{6}+{b}_{7}+{b}_{8}+{b}_{9}$	0.058**	0.066***	0.000	0.000	0.050*	0.044*
$\:{v}_{1}={b}_{1}-{b}_{2}$	0.327***	0.113*	0.075*	0.524***	0.254***	0.012
$\:{v}_{2}={b}_{3}-{b}_{4}+{b}_{5}$	-0.109*	-0.037	0.019	0.096*	0.127†	0.110
$\:{v}_{3}={b}_{6}-{b}_{7}+{b}_{8}-{b}_{9}$	0.058**	0.066***	0.000	-0.119**	-0.050*	-0.044*
Explained variance (R2 adjusted)	0.140	0.098	0.018	0.133	0.052	0.010

Note. Metaparameters$\:{u}_{1}$to$\:{v}_{3}$correspond to the linear, quadratic and cubic C-V fit ($\:{u}_{1}$to$\:{u}_{3}$) or misfit ($\:{v}_{1}$to$\:{v}_{3}$) effects. Zero values (0.000) are fixed by construction, as part of the identification strategy of the best-fitting polynomial model. Fit indices for each model are provided in Table 3.

†*p* < .10; *p < .05; **p < .01; ***p < .001.

**Fig. 4:**
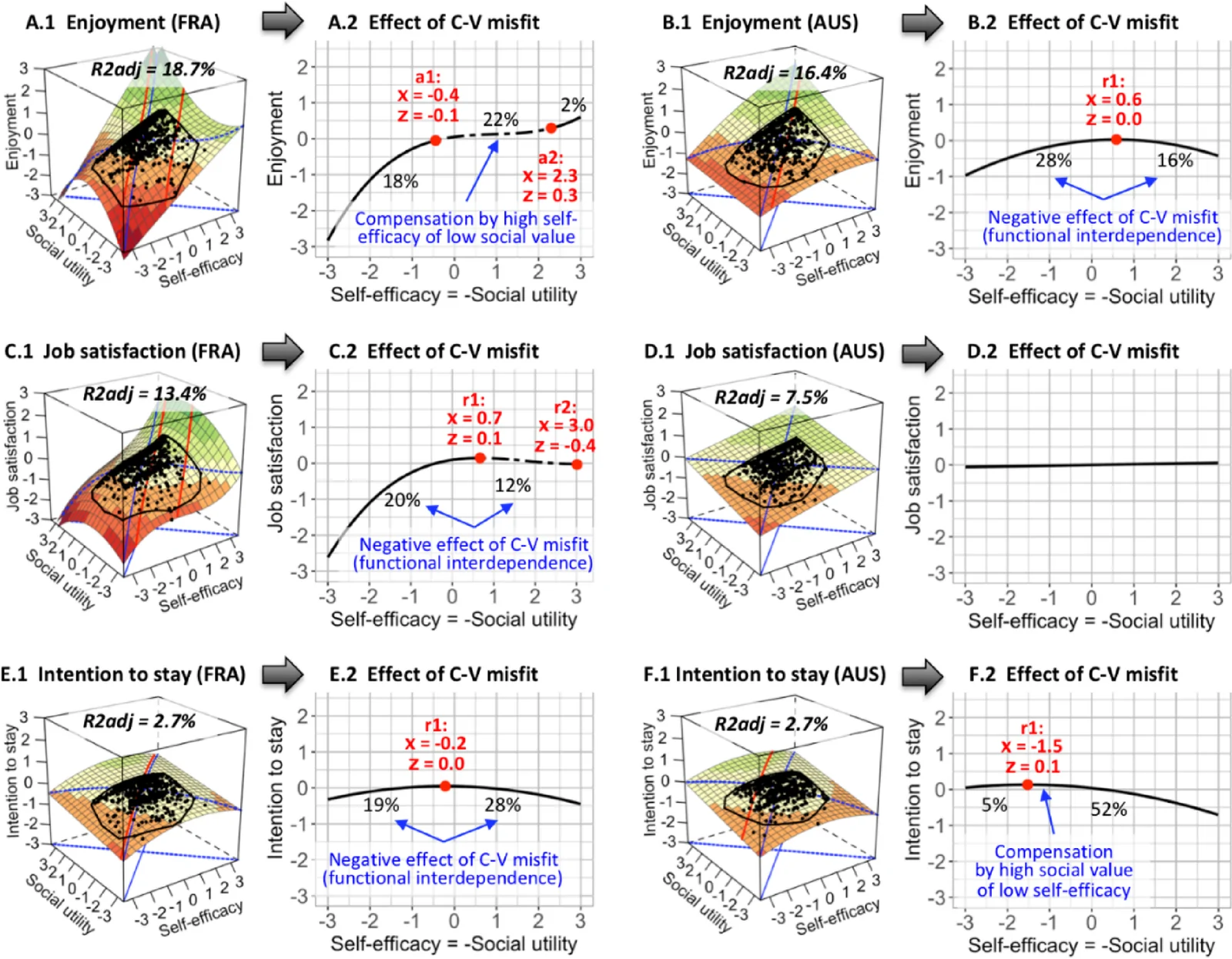
Control-value (self-efficacy*social utility) effects on teacher well-being indicators. FRA= France; AUS = Australia. Most patterns align with functional interdependence: Teacher well-being indicators peak when self-efficacy and social utility are above average (> 0), and drop when either is lacking (< 0). Other patterns of compensation by self-efficacy (A.2), functional independence (D.2), or compensation by social utility (F.2) are also observed. Fit indices and model parameters are reported in Tables 3 and 6.

For the second combination (self-efficacy*social utility), effect sizes were much larger and were meaningful for all well-being indicators. The models explained 30% to 50% more variance than the first C-V combination, underscoring the stronger statistical association between social utility value and teacher well-being (vs. personal utility value). Moreover, the response surfaces showed evidence for the comparable importance of self-efficacy and social utility value in supporting teacher well-being. The most common pattern aligned with functional interdependence (Fig. 1, C.2): Optimal levels of teacher well-being occurred when teachers experienced high and balanced amounts of C-V constructs (positive fit), but well-being dropped when either construct was low. Interdependence significantly affected job satisfaction and intention to stay for 32% to 47% of French teachers (Fig. 4, C.2 and E.2), but also teacher enjoyment for 42% of Australian teachers (Fig. 4, B.2). Another pattern showed additive, beneficial effects of self-efficacy and social utility value on job satisfaction (Australian sample, Fig. 4, D.2), characterized by a lack of interdependent or compensatory processes (Fig. 1, C.1): Job satisfaction peaked when both C-V constructs were high, but levels in one construct did not modify effects of the other construct. Finally, two asymmetric patterns appeared. In France, experiencing high self-efficacy was related to average enjoyment levels, even when teachers perceived low social utility in their job (Fig. 4, A.2), a process compatible with compensation by self-efficacy (Fig. 1, C.3). In Australia, intention to stay was positively predicted by the social value of teaching which compensated for low feelings of self-efficacy for teaching (but not conversely) (Fig. 4, F.2).

**Table 6:** Effects of control (self-efficacy) and value (social utility) on teacher well-being.

Variable	France			Australia		
	Enjoyment	Job satisfaction	Intention to stay	Enjoyment	Job satisfaction	Intention to stay
*Parameters*						
Intercept ($\:{b}_{0})$	0.051	0.094**	0.045	0.002	0.000	0.039
Self-efficacy ($\:{b}_{1})$	0.283***	0.198***	0.085***	0.304***	0.188***	0.028
Social utility ($\:{b}_{2}$)	0.125***	0.026	0.106***	0.213***	0.169***	0.154***
Self-efficacy^2 ($\:{b}_{3}$)	-0.009	-0.005	-0.048*	-0.026*	0.000	-0.041*
Self-efficacy* Social utility ($\:{b}_{4}$)	0.066†	0.058*	0.000	0.052*	0.000	0.000
Social utility^2 ($\:{b}_{5}$)	-0.054**	-0.094***	0.000	0.000	0.000	0.000
Self-efficacy^3 ($\:{b}_{6}$)	0.000	0.029***	0.000	0.000	0.000	0.000
Self-efficacy^2* Social utility ($\:{b}_{7}$)	0.000	0.000	0.000	0.000	0.000	0.000
Self-efficacy * Social utility ^2 ($\:{b}_{8}$)	0.046**	0.000	0.000	0.000	0.000	0.000
Social utility^3 ($\:{b}_{9}$)	0.000	0.000	0.000	0.000	0.000	0.000
*Metaparameters*						
$\:{u}_{1}={b}_{1}+{b}_{2}$	0.409***	0.224***	0.191***	0.516***	0.357***	0.182***
$\:{u}_{2}={b}_{3}+{b}_{4}+{b}_{5}$	0.004	-0.041	-0.048*	0.026*	0.000	-0.041*
$\:{u}_{3}={b}_{6}+{b}_{7}+{b}_{8}+{b}_{9}$	0.046**	0.029***	0.000	0.000	0.000	0.000
$\:{v}_{1}={b}_{1}-{b}_{2}$	0.158***	0.172***	-0.021	0.091*	0.020	-0.125**
$\:{v}_{2}={b}_{3}-{b}_{4}+{b}_{5}$	-0.129*	-0.157***	-0.048*	-0.078*	0.000	-0.041*
$\:{v}_{3}={b}_{6}-{b}_{7}+{b}_{8}-{b}_{9}$	0.046**	0.029***	0.000	0.000	0.000	0.000
Explained variance (R2 adjusted)	0.187	0.134	0.027	0.164	0.075	0.027

Note. Metaparameters$\:{u}_{1}$to$\:{v}_{3}$correspond to the linear, quadratic and cubic C-V fit ($\:{u}_{1}$to$\:{u}_{3}$) or misfit ($\:{v}_{1}$to$\:{v}_{3}$) effects. Zero values (0.000) are fixed by construction, as part of the identification strategy of the best-fitting polynomial model. Fit indices for each model are provided in Table 3.

†*p* < .10; *p ≤ .05; **p < .01; ***p < .001.

### Mediation by self-efficacy of needs-supplies fit effects on teacher well-being

To further probe the integrative framework, we examined to what extent N-S fit effects related to teacher well-being, and whether these effects transited through their effects on self-efficacy. In both France and Australia, complex N-S fit effects explained nontrivial proportions of variance in teacher well-being indicators (R^2^_adj_ between 2.2% and 4.5%), with a single exception for intention to stay in Australia (R^2^_adj_ = 0.5%). Response surfaces for these models (Fig. S4, SI) were consistent with N-S fit theory and the findings on self-efficacy, showing that CPL supplies were beneficial to teacher well-being only when they exceeded CPL need levels.

Mediation models further demonstrated that these N-S fit effects transited significantly via teacher self-efficacy (for details, see Section C, SI). These models explained large proportions of variance in enjoyment and job satisfaction (7% to 13%) and a nontrivial amount in intention to stay (3%). Across both contexts, all key polynomial effects defining the N-S fit solutions yielded significant indirect effects via self-efficacy, with direct effects often becoming nonsignificant at 5% and reducing in size. These findings indicate that the associations between N-S fit and teacher well-being primarily operate through teachers’ perceived control over classroom processes, consistent with the integrative framework (Fig. 1).

## Discussion

Supporting teacher well-being remains one of the most pressing challenges of contemporary educational systems. The international framework of TALIS identified teachers’ continuous professional learning (CPL) as a key lever to support student learning^18^. In this study, we expanded the potential of CPL by considering its protective role in supporting teacher well-being. We developed an integrative theoretical framework, positing that CPL may reinforce teachers’ sense of control over classroom processes insofar as supplies in CPL exceeded teachers’ needs for it (*needs-supplies fit theory*); and that higher control is associated with higher teacher well-being, particularly when it coincides with a high perceived value for the profession, e.g., its personal or social utility (*control-value theory*). This framework was tested using national-representative TALIS 2024 data from France and Australia. Results were largely consistent with the proposed framework, shedding new light on processes that may underlie teacher well-being and on strategies that could support it.

### Main findings

The first contribution of this study was showing that supplies in CPL related positively to teacher control (i.e., teaching self-efficacy), but that this effect strongly depended on their needs levels (RQ1). Aligning with N-S fit research^21,23,29^, the effects of CPL supplies alone were trivial; only by integrating CPL needs did the model contribute to explain self-efficacy levels meaningfully. This was because, in both France and Australia, the positive effects of CPL became evident when CPL supplies (i.e., the amount of CPL teachers reported having received) exceeded CPL needs (i.e., the amount of CPL teachers reported needing for professional development); conversely, when supplies were deficient relative to needs, self-efficacy levels dropped. These associations became stronger as the N-S discrepancy grew larger, significantly affecting self-efficacy levels of 1 in 2 teachers overall. Moreover, we also found that N-S fit effects did not relate to teacher value constructs. This pattern is consistent with the view that professional learning is more closely related to control appraisals (self-efficacy) than to value appraisals, the latter being shaped by broader career and identity processes such as prior teaching experiences or social influences^2,31^.

The second contribution was assessing the joint effects of teacher control and value on teacher well-being indicators including enjoyment of teaching, job satisfaction, and intention to stay in teaching^4,12,46^. Across French and Australian samples, results showed that teacher well-being peaked when teachers (1) felt in control (high self-efficacy) and, simultaneously, (2) valued their profession for its utility as a social contribution (and, to a lesser extent, for its personal utility). In other words, teacher control and value both appeared important for optimal teacher well-being, a finding consistent with C-V theory^24–26^ and related frameworks^31,41,42^. However, diverse patterns emerged when teachers were lacking either control or value. The most common aligned with functional interdependence^30,45^: low levels in one construct (e.g., low social utility value) sufficed to offset the beneficial effects of the other construct (e.g., high self-efficacy), resulting in lack of well-being (e.g., low enjoyment). This interdependence was reflected in most outcomes (enjoyment, job satisfaction) and affected one third to one half of teachers. The second pattern was a compensatory mechanism, whereby high levels in self-efficacy compensated for low levels in teacher value to maintain intermediate levels of well-being. This was most evident in C-V effects involving personal utility value, reflecting the lesser importance of personal utility (vs. social utility) in supporting teacher well-being. Taken together, the results highlight that while multiple C-V combinations may support teachers’ adaptation to some degree, the co-occurrence of strong control (self-efficacy) and value (social utility) appears the most adaptive pattern in our data.

### Limitations

Several limitations qualify the interpretation and scope of these findings. First, the study focused on secondary school teachers and does not necessarily generalize to primary school teachers. This focus was justified because the teacher attrition problem (our study’s impetus) is most pronounced among secondary school teachers^17,52^; and by the fact that our instrument for CPL needs and supplies contained several items referring to subject-matter expertise (see Table 1), more relevant to secondary school teachers.

Second, all measures relied on self-reports^53^. This approach was the only feasible for the latent constructs involved, and psychometrics showed the measures were reliable for analysis and reflected the same latent constructs in both countries (as documented by metric invariance test, *SI Text*). Nonetheless, future research could test the proposed framework using objective indicators of teacher retention or turnover, thus examining whether the observed relations translate to actual behavioral outcomes beyond intentions to stay.

Third, the data were cross-sectional, precluding causal inference; accordingly, the associations reported in this study should not be interpreted as demonstrating causal effects. Core elements of the proposed model, such as C-V processes, have been validated from a causal perspective in longitudinal studies^48,54^; CPL N-S fit processes, on the other hand, can hardly be construed as outcomes (vs. determinants) of teacher well-being. It is possible, however, that teacher control (or value) exerts a reciprocal influence on CPL needs or supplies, for example if low self-efficacy or high value trigger participation in CPL. Future studies using a longitudinal design and dedicated techniques^55,56^ could investigate reciprocal effects over time to better understand the causal ordering among these constructs.

Fourth, our results may not generalize to other contexts than France or Australia. In particular, emergent work suggests that the scope of teaching motivations differs substantially between Western and non-Western societies, as well as between regions with high versus low human development indices (HDI). Specifically, teachers in Western and high-HDI countries report lower personal utility value for entering a teaching career (e.g., lower job benefits), compared to teachers from other regions^2^. Such regional differences in teacher value may modify processes of functional interdependence or compensation between C-V constructs, by changing the order of importance of value types (e.g., personal vs. social utility). Similarly, CPL formats may differ markedly from those in France and Australia. For these reasons, future international comparisons are warranted to explore the generalizability and specificities of N-S fit and C-V effects found in our study.

### Implications

The present findings provide a coherent picture for understanding and supporting teacher well-being via professional learning (CPL). From a theoretical perspective, they provide first-hand evidence for the proposed integrative framework linking N-S fit theory and C-V theory (Fig. 1). In this model, CPL can be construed as a lever for teacher control, in that higher CPL supplies relative to needs are associated with higher self-efficacy beliefs. However, in line with our findings, such enhanced control appears to relate to improved teacher well-being (i.e., greater enjoyment, job satisfaction, and intention to stay) primarily when accompanied by high levels of teacher value, particularly appraisals that teaching is of social value. This means that increasing teacher control in isolation (via CPL N-S fit processes) might be insufficient to reverse patterns of reduced motivation and foster teacher well-being, if no changes are made to acknowledge and reinforce the social value of teaching. In a nutshell: “Teachers need more than knowledge”^30^; they also need to “achieve their positive motivations to teach”^31^.

From a more practical perspective, the evidence on N-S fit effects points to a fundamental principle of person-environment fit theory^21,23,29^: There appears to be no absolute quantity of CPL that guarantees improvement in self-efficacy; its apparent benefits seem to depend on how well it exceeds individuals’ requirements. Moderate amounts of CPL may therefore suffice for some teachers with low professional learning needs at present (moderate supplies > low needs), but the same amounts may be ineffective or even detrimental for others facing greater challenges (moderate supplies < high needs). For educational systems, this has several implications. First, in order to allow teachers to reach high levels of CPL supplies, policies should strive to promote diversified and easily accessible CPL formats. In the present study, for instance, teachers reported stronger CPL supplies in France compared with Australia, and this difference was paralleled by their greater participation and wider range of CPL formats (e.g., qualification programs, mentoring, teacher networks, visits to other schools, education conferences or seminars, etc.; see SM-A). This suggests that greater CPL opportunities translate to greater CPL supplies. Second, professional learning should be flexible enough to match individual differences in needs. As underscored in TALIS^18^, teachers may experience changes in their CPL needs due to incorporating new technologies, or addressing new behaviors or norms across generations of students. At the same time, teachers may be differentially equipped due to unequal initial teacher education opportunities. These intra- and interindividual differences may contribute to large differences in CPL needs. Arguably, CPL supplies should be tailored and distributed according to these differences, as opposed to being uniformly implemented (or imposed) for all teachers.

According to our framework, supporting teachers’ self-efficacy through well-designed CPL may not be sufficient on its own to foster teacher well-being. Indeed, this must be complemented by policies that also support teachers’ valuing of the profession. This highlights another major implication for both research and policymaking. Although teacher value processes—particularly social utility value—have received much attention in relation to career determination processes^31,41^, this factor has received less attention in relation to teacher wellbeing. Wellbeing studies have focused on teachers’ personality traits, coping resources, job competencies, and intrinsic motivation for teaching^57^. Future research should further explore, for example, how specific value types (e.g., social vs. personal utility) interact with self-efficacy to foster well-being and prevent teacher attrition. At the societal level, initiatives should be undertaken to revalue the teaching profession, such as emphasizing teachers’ impact on future generations, fostering public recognition, and improving job conditions. Such initiatives could help support or even restore teachers’ positive appraisals of their profession, and may contribute to reversing the “teaching motivation paradox” in Western societies, where high vocational calling remains but extrinsic incentives have declined^2^. The present findings showing limited associations between CPL and teacher value also raise the question of how such associations might be reinforced. For example, CPL formats could be designed to reflect on the social contribution of teaching, notably via momentary or targeted tutoring with students who experience disadvantage or various disabilities. Such formats could create opportunities to “rekindle” the vocational call to teach and reconnect teachers with a sense of purpose^31,58^.

Taken together, the findings are consistent with the integrated framework (Fig. 1) proposing that CPL is associated with higher teacher well-being when it supports teachers’ sense of control by addressing their training needs, and when control coincides with high valuing of the profession. These structural conditions, observed consistently across France and Australia, provide a coherent account of how professional learning, motivational appraisals, and well-being are interlinked. By identifying the specific combinations of CPL supplies, control, and value that are linked to higher well-being, the study points to actionable levers that educational systems could consider when confronting rising teacher attrition.

## Method

### Participants

Participants were lower secondary (ISCED 2) teachers from France and Australia who took part in the OECD TALIS 2024 framework^18^. Both countries are long-standing TALIS participants and have consistently provided high-quality, nationally representative data on teachers’ professional learning and working conditions^14,59^. In TALIS 2024, each country implemented a two-stage stratified probability sampling design. Schools were first selected with probability proportional to size, and teachers were then randomly sampled within each participating school. This design ensured representativeness of the teaching workforce. More specifically, the French sample included 2877 teachers (age: <25 = 10.9%, 25–29 = 21.9%, 30–39 = 33.0%, 40–49 = 29.2%, ≥ 50 = 4.9%); 57% were female and 15% had less than 5 years of teaching experience. The Australian sample comprised 3035 teachers (age: <25 = 17.8%, 25–29 = 26.7%, 30–39 = 26.2%, 40–49 = 22.3%, ≥ 50 = 7.1%), including 61% female and 22% having less than 5 years of experience. Detailed demographics and sampling procedures are available from TALIS reports^14^.

### Measures

All measures are from teacher self-reports obtained through the 82-item TALIS Teacher Questionnaire. The questionnaire was completed online (sometimes in paper versions), taking 45–60 min on average to complete^14^. For each construct in our model (Fig. 1), teachers answered multiple items (except for intention to stay, assessed through a single item) reflecting their perceptions of professional learning, teacher control and value, and teacher well-being. To retain only true-score variance, scores were extracted from multi-item confirmatory factor analytic models demonstrating excellent fit to the data (e.g., CFI ≥ 0.950, RMSEA ≤ 0.060) and high internal consistency ($\:\omega\:\ge\:.70$), thereby obtaining reliable, error-free measures. These models were validated for metric invariance across countries, ensuring that the constructs were commensurate across countries and that any observed differences reflected substantive processes rather than measurement artefacts. Details on item content, response scales, construct validity, metric invariance, and reliability indices for each measure are provided in the *SI Text* (Tables S1 to S3).

#### CPL needs and supplies

Teachers’ *CPL needs* and *CPL supplies* were assessed using parallel sets of 15 items relating to their professional skills (e.g., “The pedagogy of the subject matter I teach”, “Classroom management for student behavior”; see also Table 1). The TALIS 2024 version incorporates revisions that respond to Meroño et al.’s critique of earlier versions, by strengthening the coverage of pedagogical, social, and digital competences, to obtain a more balanced and updated assessment of teachers’ CPL. Aligning “atomistic” measures of N-S fit processes^21,23,29^, both sets share identical content domains to enable a comparison of CPL needs versus supplies. For needs, teachers rated the extent to which they needed CPL in the domain tapped by each item on a 4-point scale (4 = no need at all, 1 = high level of need, reversed-coded in our study). For supplies, they indicated whether their current learning activities had included this domain (0 = No, 1 = Yes), yielding a sum score of supplies. Although rating scales differed, the latent constructs were commensurate in the standardized metric (M = 0, SD = 1). Both scales demonstrated high internal consistency ($\:{\omega\:}_{FRA}=.881,{\omega\:}_{AUS}=.899\:$for needs; $\:{\omega\:}_{FRA}=.821,{\omega\:}_{AUS}=.802$ for supplies) and correlated weakly (Table 2), supporting their use as parallel indicators of needs-supplies fit processes.

#### Teacher control: self-efficacy

Consistent with C-V theory^26,30^, teacher control was measured through teachers’ perceived *self-efficacy* to influence classroom processes, including the ability to manage the classroom (e.g., “Control disruptive behavior in class”), to engage students in learning (e.g., “Motivate students who show low interest in school work”), and to adapt instruction to diverse learning needs (e.g., “Vary instructional strategies in my classroom”)^18,43^. The TALIS scale comprises 12 items rated on a 4-point scale (1 = not at all, 4 = a lot) and demonstrated excellent internal consistency ($\:{\omega\:}_{FRA}=.889,{\omega\:}_{AUS}=.907$).

#### Teacher value: Personal and social utility

Aligning with C-V theory and related theories^42^, teacher value was operationalized in TALIS through items drawing from the Factors Influencing Teaching (FIT-) Choice framework^41^. In TALIS, these items are rated on a 4-point scale (1 = not important at all, 4 = very important), measuring *personal utility value* reflecting job benefits (3 items, e.g., “Teaching is a secure job”) and *social utility value* reflecting its contribution to society (3 items, e.g., “Teaching makes a worthwhile social contribution”). These two factors yielded satisfactory internal consistency ($\:{\omega\:}_{FRA}=.693,{\omega\:}_{AUS}=.761$ for personal utility; $\:{\omega\:}_{FRA}=.779,{\omega\:}_{AUS}=.791$ for social utility) and showed low or moderate correlations ($\:{r}_{FRA}=.089,{r}_{AUS}=.200$). Given their modest correlation, they were retained as distinct facets of teacher value.

#### Enjoyment

Based on C-V theory and the Teacher Emotions Scale^44^, teacher *enjoyment* was assessed as a positive activating emotion, via 4 items reflecting feelings of enthusiasm, pleasure, and satisfaction when teaching (e.g., “I often feel happy while I teach”, “The interesting challenges of teaching give me satisfaction”). Each item was rated on a 4-point scale (1 = never or almost never, 4 = always or almost always). The 4-item scale showed excellent reliability ($\:{\omega\:}_{FRA}=.841,{\omega\:}_{AUS}=.824$).

#### Job satisfaction

Teachers were asked to rate their degree of satisfaction with the teaching profession (e.g., “All in all, I am satisfied with my job”), including their current working environment (e.g., “I enjoy working at this school”), via 10 items rated on a 4-point scale of agreement (1 = strongly disagree, 4 = strongly agree). The scale showed high internal consistency in both samples ($\:{\omega\:}_{FRA}=.854,{\omega\:}_{AUS}=.879$).

#### Intention to stay

To capture teachers’ intention to remain in the profession, we constructed a continuous indicator reflecting the number of years envisaged, relative to the remaining career span. Indeed, in TALIS, teachers are asked how many more years they envisage staying in teaching. Yet, the raw answers they provide may reflect differences in career lifespan; for instance, teachers near retirement have limited years left, as compared to early career teachers. To adjust for this, we built a career lifespan indicator by subtracting respondents’ age (i.e., the midpoint of age groups only available in TALIS: 1 = under 25, 2 = 25–29, 3 = 30–39, 4 = 40–49, 5 = 50 and above) from a reference retirement age of 65. The *intention to stay* score was then calculated by dividing the envisaged number of years in teaching (raw values) by the career lifespan indicator: $\:intention\:to\:stay=\:years\:envisaged/(65-age\:midpoint)$. Values above 1 or below 0 (artifacts of using midpoint ages instead of actual ages) were recoded to 1, and 0, respectively. This resulted in a bounded, interpretable indicator ranging from 0 (no intention to stay) to 1 (intention to stay until retirement).

### Analytic strategy

#### Response surface analysis

All analyses were conducted using RSA based on cubic polynomial modeling^39,40^. RSA aims to obtain the best representation of how two predictors (* x* and * y*) combine in their effects on an outcome variable (* z*), by (1) specifying a polynomial model of linear or nonlinear effects occurring on the lines of fit (* x=y*, or line of congruence—LOC) and misfit (* x=-y*, or line of incongruence—LOIC), and then (2) characterizing the resulting response surface in terms of thresholds of acceleration or reversal in the curvature (for nonlinear effects) or in terms of relative levels of the predictors along the LOC and LOIC (for linear effects). This approach avoids the limitations of moderation models, which rely on an interaction term (i.e., the x*y product) that mathematically forces the LOC and LOIC to have opposite quadratic curvatures—one concave, the other convex—thereby distorting the underlying interactive processes (for an illustration, see Fig. S2, *SI Text*). By contrast, RSA lets the curvature emerge directly from the data.

Aligning with our integrative framework (Fig. 1), we used RSA to characterize how CPL needs (* x*) and CPL supplies (* y*) jointly predicted control and value outcomes (* z*); and how teacher control (* x*) and value (* y*) jointly predicted teacher well-being outcomes (* z*). To identify the most valid representation of these processes, our specification models were obtained based on the comparison of 37 families of polynomial models (FMs)^40^, as implemented in the R package *RSAtools*^60^ . All polynomial families are derived from the saturated cubic polynomial:

$$\:z={b}_{0}+{b}_{1}x+{b}_{2}y+{b}_{3}{x}^{2}+{b}_{4}xy+{b}_{5}{y}^{2}+{b}_{6}{x}^{3}+{b}_{7}{x}^{2}y+{b}_{8}x{y}^{2}+{b}_{9}{y}^{3}.$$

where $\:{b}_{0}$ is an intercept, $\:{b}_{1}\:$to $\:{b}_{9}$ are the predictor effects that determine the trends (linear effects $\:{b}_{1}$ to $\:{b}_{2}$), curvatures (quadratic and interactive effects $\:{b}_{3}$ to $\:{b}_{5}$), and asymmetric curvatures (cubic and interactive-quadratic effects $\:{b}_{6}$ to $\:{b}_{9}$) of the response surface. On this basis, polynomial families are defined by constraining selected parameters to zero (e.g., $\:{b}_{6}$=$\:{b}_{7}={b}_{8}={b}_{9}=0$, for quadratic patterns) or to equality (e.g., $\:{b}_{6}=-\frac{{b}_{7}}{3}=\frac{{b}_{8}}{3}=-{b}_{9}$, for the family of asymmetric congruence effect, FM20)^39,40^. Additive models are nested within this structure when the higher-order terms ($\:{b}_{3}\:$to $\:{b}_{9}$) are constrained to zero. A total of 37 polynomial families are compared, with characteristic response surfaces positing different kinds of linear, quadratic (U-shaped or inverted-U-shaped), or cubic (S-shaped) effects of fit, misfit, or both fit or misfit. We retained the polynomial family that best balanced model parsimony and fit to the data, using several criteria including providing the highest probability of reflecting the data generation process (i.e., highest Akaike weight—wAIC) and the highest explained variance (R2 adjusted for model complexity—R^2^_adj_), showing excellent fit to the data (CFI ≥ 0.95, RMSEA ≤ 0.06, SRMR ≤ 0.08), and displaying statistically significant higher-order effects (Wald test, * p<.05*)^39,61,62,40^. All final models met these criteria (see Table 3). Following conventional effect sizes in the behavioral sciences, explained variance values were interpreted as small/trivial (R^2^_adj_ = 0.02), medium (R^2^_adj_ = 0.13) and large (R^2^_adj_ = 0.26)^63^.

Once a best-fitting polynomial model was identified and showed nontrivial effect size (R^2^_adj_ > 0.02), we projected the corresponding response surface and examined its shape (see Figs. 2, 3 and 4). For visual clarity, 1000 random data points were projected instead of the full samples. Aligning with standard RSA methodology^38,39^, we focused on the response curve occurring over the line of misfit (* x=-y*), thereby characterizing how discrepant levels in needs and supplies ($\:CPL\:needs=-CPL\:supplies$) or control and value processes (i.e., * control=-value*) related to the outcomes of interest. For increased precision and to gauge the impact of nonlinearities, we relied on the identification of reversal point “r” where the misfit effect changed sign ($\:\frac{\partial\:z}{\partial\:x}$ = 0, e.g., from positive to negative), and of acceleration points “a” where the misfit effect reached a non-trivial size (i.e., $\:\left|\frac{\partial\:z}{\partial\:x}\right|$ = 0.30)^63,40^. To test that these curvatures were statistically meaningful rather than artefactual extrapolations of nonlinear effects, we applied a range-checking procedure adapted from the cubic RSA literature^39,40^. For each teacher datapoint situated beyond the margin of acceleration or reversal, we compared the observed outcome value with the model-predicted value and its one-tailed 95% confidence interval at the margin. When the outcome value lay outside this confidence interval and was consistent with the direction of nonlinearity (e.g., lower value when the reversal was negative, higher value when the acceleration was positive), the individual was considered affected by the nonlinearity. This enabled assessing the percentage of teachers significantly affected by reversal or acceleration.

#### Mediation analyses

Indirect effects of N-S fit on teacher well-being were estimated using the mediated polynomial regression approach described by Edwards^64^, extended here from quadratic to cubic polynomials to match our models. In this approach, indirect (mediation) effects were computed as the product of each target polynomial effect from the N-S fit model (e.g., linear, quadratic, or cubic terms involving CPL needs and supplies) and the regression coefficient linking self-efficacy to the outcome variable. This approach constitutes an approximation because it assumes self-efficacy relates linearly to teacher well-being and does not interact with value constructs, whereas our results showed instead nonlinear and interactive effects (Tables 5 and 6). This simplification was necessary because, to date, no statistical procedure exists for estimating mediation between two cubic polynomial models simultaneously. Accordingly, the mediation estimates should be interpreted as conservative approximations of the true indirect processes, possibly underestimating their magnitude.

#### Model estimation

Prior to analyses, we standardized all variables to zero means and unit variances to prevent parametric bias and facilitate interpretation and comparisons across countries. Alternative approaches (e.g., grand-mean centering, nonstandardization) can create spurious curvatures and artificially inflate between-sample differences due to unequal distributions, especially since parametric constraints placed on polynomial families assume even metric sizes for the predictors. Our standardization procedure also facilitates the interpretation of nonlinear effects by expressing the misfit axis in standardized units; this way, discrepancies between predictors (* x=-y=0.5*) correspond to deviations from the sample means in standardized units ($\:x=+0.5\:SD$, * y=-0.5SD*, representing a $\:\varDelta\:\left(y-x\right)=1SD$ relative difference).

In terms of estimator, all models used full information maximum likelihood (FIML) with robustness corrections against deviations from normality and data clustering^65,66^. FIML is most effective to reduce parametric bias arising from missing data. In our analyses, FIML accounted for 97% to 99% of French teachers and 99% of Australian teachers, thus reflecting the full national samples. Although sampling weights were tested, these created convergence issues when combined with FIML. Sensitivity checks using sampling weights with listwise deletion yielded substantively the same results, although reducing sample sizes considerably. Accordingly, the FIML-based solutions are reported as the main analyses, under the assumption that results generalize to the population-weighted estimates. Unless indicated otherwise, all tests are two-tailed. Full model outputs (parameter estimates, SEs, Wald tests, exact *p*-values, and 95% CIs) reported in the manuscript are available in the *SI Statistics* sheets.

### Ethics statement

This study used secondary anonymized data from the OECD TALIS 2024. Data collection was conducted by national TALIS centers under the oversight of the OECD TALIS Governing Board and in accordance with the ethical and legal requirements applicable in each participating country. Informed consent was obtained from all participants prior to data collection, and all methods were carried out in accordance with relevant guidelines and regulations. Because the present study relied exclusively on fully anonymized secondary-use data, no additional ethical approval was required for the analyses reported here.

## Supplementary Information

Below is the link to the electronic supplementary material.

Supplementary Material 1: SI Text

Supplementary Material 2: SI Statistics

## Data Availability

The datasets used in this study are freely available from the TALIS depository: [https://www.oecd.org/en/data/datasets/talis-2024-database.html - manuals]. TALIS microdata are subject to OECD restricted-use conditions and cannot be shared. Synthetic derived variables used for model evaluation (e.g. factor scores), as well as R scripts used for response surface modeling, are made available on the following OSF repository for reproducible results: [https://osf.io/j756k]).
