# Supplementary material for: Professional learning supports teacher well-being through needs-supplies fit and control-value processes
Source: Sci Rep. 2026 Aug 20;16:26198. doi: 10.1038/s41598-026-57946-9 (PMC13493979; doi:10.1038/s41598-026-57946-9)
Supplement: Supplementary file 1 [file 41598_2026_57946_MOESM1_ESM.pdf]

Professional Learning Supports Teacher Well-Being Through Needs-Supplies Fit and Control-Value Processes

SUPPORTING INFORMATION: *SI Text*

A. Details on Study Variables 2

Types of CPL activities and CPL Needs-Supplies Scales *2*

Psychometric Validation of Study Variables *5*

B. Details on RSA Methodology 7

Limitations of Moderation Analyses *7*

Cubic Response Surface Analysis *8*

C. Complementary Results on Response Surface Analyses 10

D. Mediation Analyses........ 13

References in Supporting Information........ 16

***Nota bene*: Additional Tables S6 to S23 are available on the *SI Statistics* sheets.**

A. Details on Study Variables

Types of CPL activities and CPL Needs-Supplies Scales

Fig. S1 Participation to different types of continuous professional learning (CPL) activities

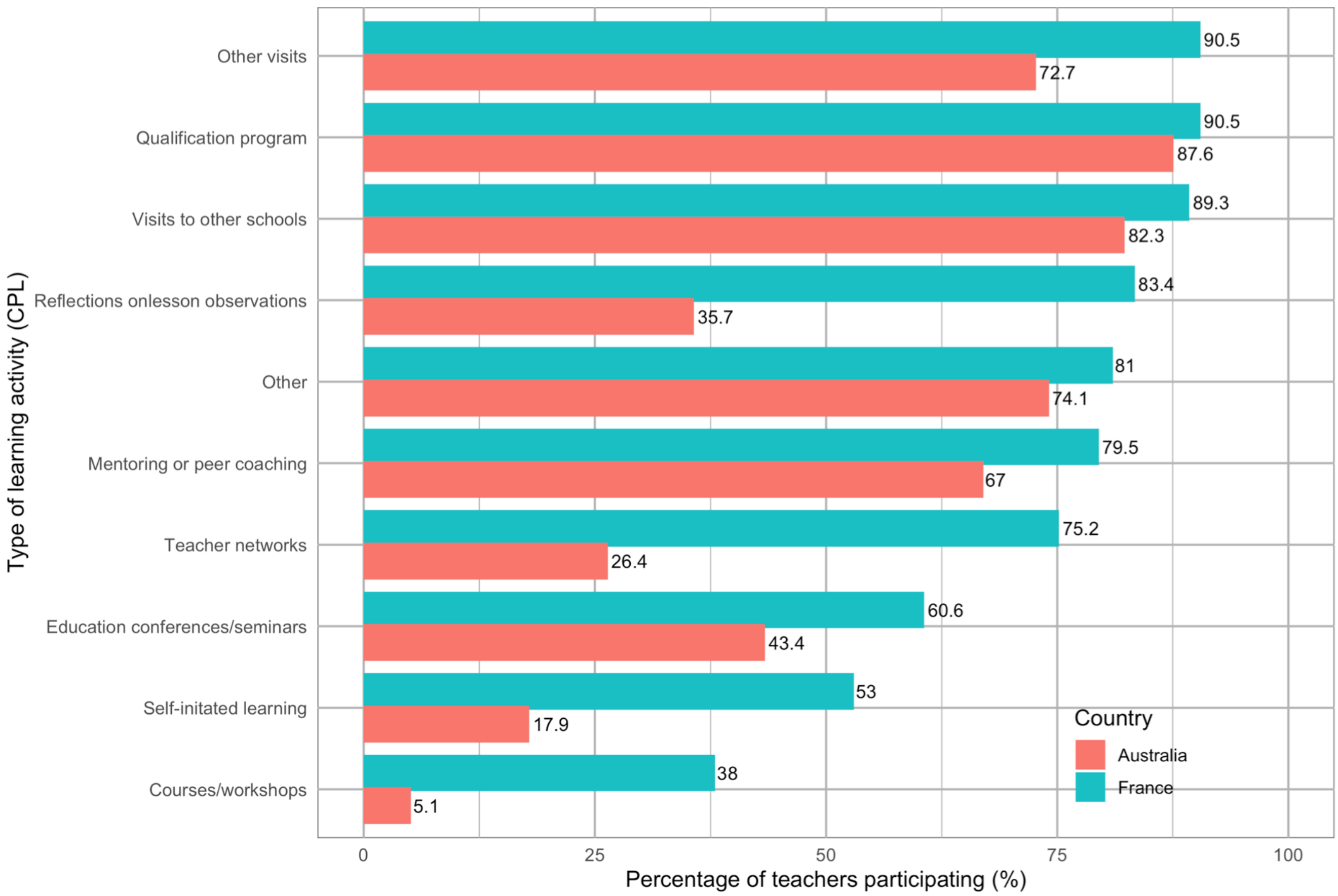

Table S1. Item Wordings and Response Scale in the Teacher Questionnaire of TALIS 2024

Variable	Item stem and response scale	Items
CPL Needs	For each of the areas listed below, indicate the extent to which you currently need professional learning activities.<br>4-point scale: 1=no need at present to 4=high level of need.	Knowledge and understanding of my subject field(s);<br>The pedagogy of the subject matter(s) I teach;<br>Knowledge of the curriculum;<br>Student assessment practices;<br>Pedagogical skills for incorporating digital resources;<br>Technical skills for digital resources;<br>Skills for using artificial intelligence;<br>Classroom management for student behaviour;<br>School management and administration;<br>Approaches to individualised learning;<br>Teaching students with special education needs;<br>Teaching in multicultural or multilingual setting;<br>Analysis and use of student assessments;<br>Teacher-parent/guardian co-operation;<br>Methods for supporting students' social and emotional learning
CPL Supplies	Were any of the following topics included in your professional learning activities during the last 12 months?<br>Binary scale: 0=No, 1=Yes.	Same items as CPL Needs
Self-efficacy	In your teaching, to what extent can you do the following? 4-point scale: 1=not at all to 4=a lot.	Get students to believe they can do well;<br>Help students value learning;<br>Craft good questions;<br>Control disruptive behaviour;<br>Motivate low-interest students;<br>Make expectations clear;<br>Help students think critically;<br>Get students to follow rules;<br>Calm a disruptive student;<br>Use various assessment strategies;<br>Provide alternative explanations;<br>Vary instructional strategies
Personal Utility	How important are the following factors for you as a teacher? 4-point scale: 1=not important at all to 4=of high importance.	Teaching is a secure job;<br>Working hours fit with family responsibilities;<br>Commitment flexibility;<br>I like working with children/adolescents
Social Utility	How important are the following factors for you as a teacher? 4-point scale: 1=not important at all to 4=of high importance.	Influence the next generation;<br>Work against social disadvantage;<br>Worthwhile social contribution
Enjoyment	How strongly do you agree or disagree with these statements	I often feel happy while I teach;<br>I teach with enthusiasm;

Variable	Item stem and response scale	Items
	about your experiences as a teacher? 4-point scale: 1=strongly disagree to 4=strongly agree.	Teaching challenges give me satisfaction; I like the subjects I teach
Job Satisfaction	How strongly do you agree or disagree with the following statements? 4-point scale: 1=strongly disagree to 4=strongly agree.	Advantages outweigh disadvantages; Would choose teaching again; Would change schools (rev.); Regret becoming a teacher (rev.); I enjoy working at this school; Wonder about choosing another profession (rev.); Recommend this school; Teaching valued in society; Satisfied with performance; All in all satisfied with job
Intention to Stay	How many more years do you want to continue to work as a teacher? Numeric input (nbr of years)	Computed score: years envisaged/(65 - age midpoint).

Psychometric Validation of Study Variables

Psychometric analyses were conducted to validate the measurement structure of all study variables in France and Australia. First, we estimated confirmatory factor analysis (CFA) models to verify that each scale coherently captured its intended construct. Unidimensional constructs were tested using single-factor CFAs; multidimensional constructs were evaluated using bifactor CFAs to isolate the general latent dimension underlying all items while accounting for domain-specific variance. For example, for CPL needs and CPL supplies, the same bifactor structure was applied, comprising a general factor (needs or supplies) and specific factors aligned with the substantive domains of CPL. Across variables and countries, model fit indices were excellent and internal consistency estimates were satisfactory (Table S2).

Second, we examined the comparability of factor structures across France and Australia. Metric invariance was evaluated by contrasting a configural multigroup solution, in which factor loadings were freely estimated across countries, with a metric solution constraining loadings to equality. Metric invariance is sufficient to justify the extraction of commensurate factor scores and thus to ensure that any observed cross-national differences reflect substantive variation rather than measurement artefacts. In line with established guidelines, models were considered invariant when overall fit met conventional thresholds (CFI $\geq$ .90, RMSEA $\leq$ .08) and when changes between configural and metric models remained within recommended limits (ΔCFI $\geq$ –.01, ΔRMSEA $\leq$ .015) [1–3]. All constructs satisfied these criteria; for enjoyment, invariance was achieved after freely estimating one item loading (out of 4), yielding a partial (but predominantly invariant) solution.

Together, these analyses show that the instruments exhibited strong psychometric properties, were reliably measured in each country, and were commensurate across France and

Australia. This provides confidence that the patterns of N-S fit and C-V effects reported in the main text reflect substantive processes in psychological functioning rather than idiosyncrasies in scale functioning.

Table S2. Fit Indices of Measurement Models and Construct Reliability

	chi2	DF	CFI	TLI	RMSEA	SRMR	Omega (reliability)
France							
CPL Needs	575.8	75	0.948	0.928	0.064	0.040	0.881
CPL Supplies	280.7	75	0.972	0.960	0.035	0.028	0.821
Self-efficacy	329.9	42	0.974	0.959	0.054	0.024	0.889
Personal Utility	54.5	9	0.977	0.962	0.060	0.054	0.693
Social Utility	54.5	9	0.977	0.962	0.060	0.054	0.779
Enjoyment	5.9	2	0.998	0.995	0.036	0.008	0.841
Job Satisfaction	293.1	27	0.970	0.950	0.065	0.037	0.854
Australia							
CPL Needs	433.2	75	0.966	0.952	0.056	0.034	0.899
CPL Supplies	380.1	75	0.963	0.948	0.040	0.030	0.802
Self-efficacy	482.8	42	0.964	0.943	0.068	0.034	0.907
Personal Utility	111.2	9	0.952	0.921	0.094	0.093	0.761
Social Utility	111.2	9	0.952	0.921	0.094	0.093	0.791
Enjoyment	5.5	2	0.998	0.994	0.038	0.009	0.824
Job Satisfaction	240.6	27	0.977	0.962	0.061	0.026	0.879

Note. Teacher value constructs are specified within the same CFA.

Table S3. Metric Invariance Testing Across French and Australian Samples

	chi2	DF	CFI (ΔCFI)	TLI	RMSEA (ΔRMSEA)	SRMR
CPL Needs	1127.1	174	0.953 (-0.005)	0.944	0.059 (-0.001)	0.044
CPL Supplies	1138.1	174	0.938* (-0.029)	0.925	0.048 (0.010)	0.057
Self-efficacy	948.6	104	0.964 (-0.004)	0.954	0.059 (-0.002)	0.036
Personal and social utility	175.8	22	0.962 (-0.002)	0.948	0.073 (-0.005)	0.075
Enjoyment	20.8	6	0.997 (-0.001)	0.993	0.042 (0.005)	0.017
Job Satisfaction	690.0	69	0.964 (-0.01)	0.953	0.065 (0.002)	0.050

Note. Values correspond to the metric solution in which factor loadings are constrained to equality across samples; values in parentheses indicate the changes relative to the configural solution.

B. Details on RSA Methodology

Limitations of Moderation Analyses

Complex interaction patterns are usually investigated using moderation analyses, in which the main effects of predictors such as CPL needs (x) and CPL supplies (y) are allowed to vary as a function of their interaction term (the product of needs*supplies). In our case study, this approach can show, for instance, that high levels of CPL supplies help produce higher teacher control or value when they match high CPL needs, illustrating a needs-supplies fit effect (Fig. S2, panel A). Although widely used, this method imposes strong structural constraints with two major (sometimes negative) consequences. First, because the predictors can be rewritten as functions of one another along the lines of congruence (LOC, where $x = y$) and incongruence (LOIC, where $x = -y$), the interaction term automatically induces a quadratic effect along both lines. These nonlinearities remain hidden in standard 2D projections of moderation models, leaving key processes unattended; for example, potential negative effects of excessive supplies or accelerating benefits of strong N-S alignment (Fig. S2, panel B). Second, enforcing the same quadratic form along the LOC and LOIC can distort the true response surface when the underlying patterns differ, such as when a cubic trend is present along the LOIC and a linear trend along the LOC. Such distortions can alter the substantive interpretation, for instance by obscuring an acceleration of positive effects at excessive supplies or by masking a linear, rather than nonlinear, effect of N-S fit (Fig. S2, panel C). This example illustrates the limitations of moderation analyses: Relevant nonlinearities may be overlooked, and spurious patterns may emerge from the imposed structure. Similar issues have been documented in other applications as well. [4,5]

Fig. S2 Example of potential hidden or spurious processes in moderation analyses

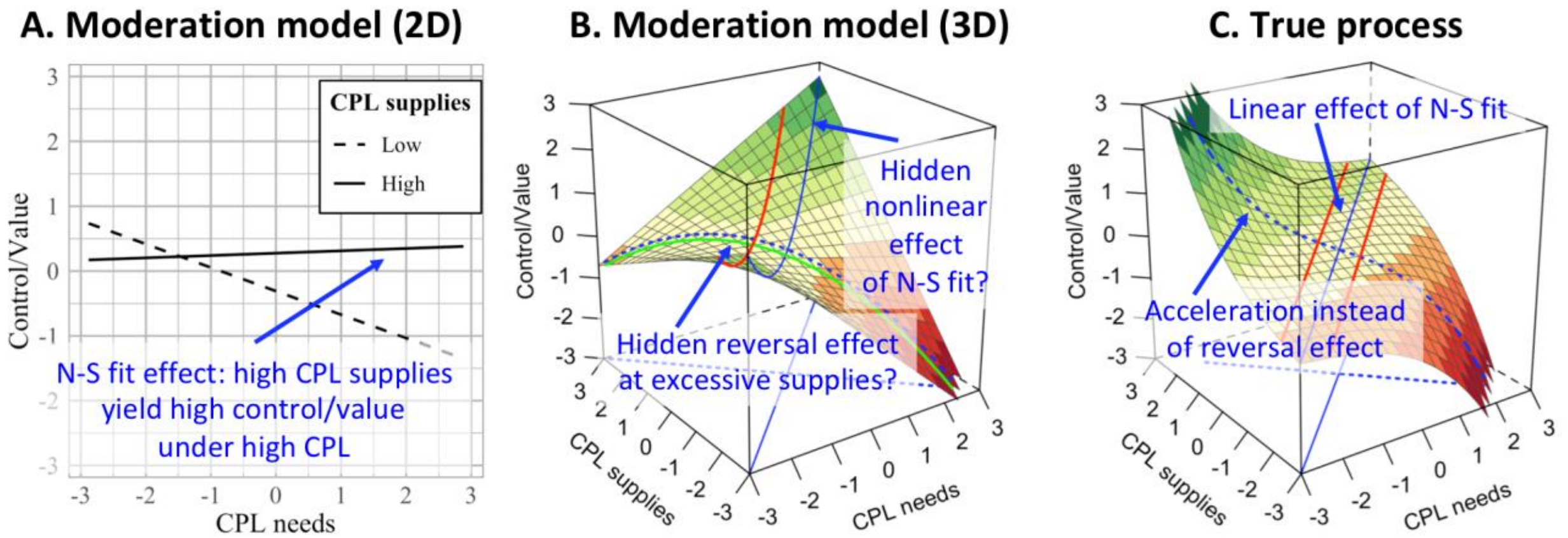

Note: Response surface simulated using CPL needs (x) and CPL supplies (y). Because x and y are equivalent along the line of fit ($x = y$) and the line of misfit ($x = -y$), the interaction term ($x * y$) induces a quadratic form on both lines, with opposite curvature. The resulting surface is therefore constrained to bend in opposite directions on the LOC and LOIC. These nonlinearities are not visible in conventional 2D moderation plots (panel A) and may obscure meaningful accelerations or reversals in teacher control/value as needs and supplies combine (panel B). More generally, this imposed quadratic structure is highly restrictive and may fail to represent the underlying process, for example by contradicting cubic or linear effect on both lines (panel C). Cubic RSA, as used in this study, avoids this limitation by allowing linear, quadratic, or cubic shapes to emerge independently along the LOC and LOIC.

Cubic Response Surface Analysis

Instead of moderation analysis, we used cubic response surface analysis (RSA), a method designed to model how two predictors (here, $x = needs$ and $y = supplies$) interact to shape a third variable ($z = self\text{-}efficacy$). The cubic polynomial model $z = b_0 + b_1x + b_2y + b_3x^2 + b_4xy + b_5y^2 + b_6x^3 + b_7x^2y + b_8xy^2 + b_9y^3$ decompose the response *z* into first-order (b_1 to b_2), second-order (b_3 to b_5), and third-order (b_6 to b_9) effects of *x* and *y*. These effects correspond respectively to the linear trends, curvature, and asymmetric curvatures that together define the shape of the response surface *z*. The model can reproduce a wide variety of theoretically meaningful response shapes, depending on how these parameters are configured.

To organize and test these patterns systematically, we adopted a comparative strategy that is equivalent to selecting the best-fitting model among thousands of possible cubic polynomials. This strategy is based on the classification of models into families of polynomial models (FM, 37 to date), each defined by a distinct set of equality or zero constraints applied to the parameters above [6–8]. Each family corresponds to a canonical response surface that captures specific interactive processes—such as rising ridges, symmetric or asymmetric fit, and threshold reversals—while reducing the complexity of unconstrained estimation. A comparative framework contrasting competing families to identify best-fitting solutions is therefore warranted to establish whether linear, quadratic, or cubic patterns characterize the data structure. The best-fitting FM was selected based on several information-theoretic criteria and statistical tests [9]. These included: passing the tests of absolute fit against the saturated cubic model to ensure no relevant information is lost [10]; having a high AIC/BIC weights to select obtain a parsimonious solution [11,12] and, simultaneously, a high explained variance to enhance replicability of the solution [13]; and, finally, showing excellent fit to the data (e.g., $CFI \geq 0.95$, $TLI \geq 0.95$, $RMSEA \leq 0.06$, $SRMR \leq 0.08$) [14,15]. Following guidelines in behavioral sciences [16,17], the contribution of D-R fit processes to the explained variance in outcomes was interpreted using standard effect size benchmarks: small (R2 ≥ .02), medium (R2 ≥ .13), and large (R2 ≥ .26).

C. Complementary Results on Response Surface Analyses

Fig. S3 Complementary response surfaces for negligible effects

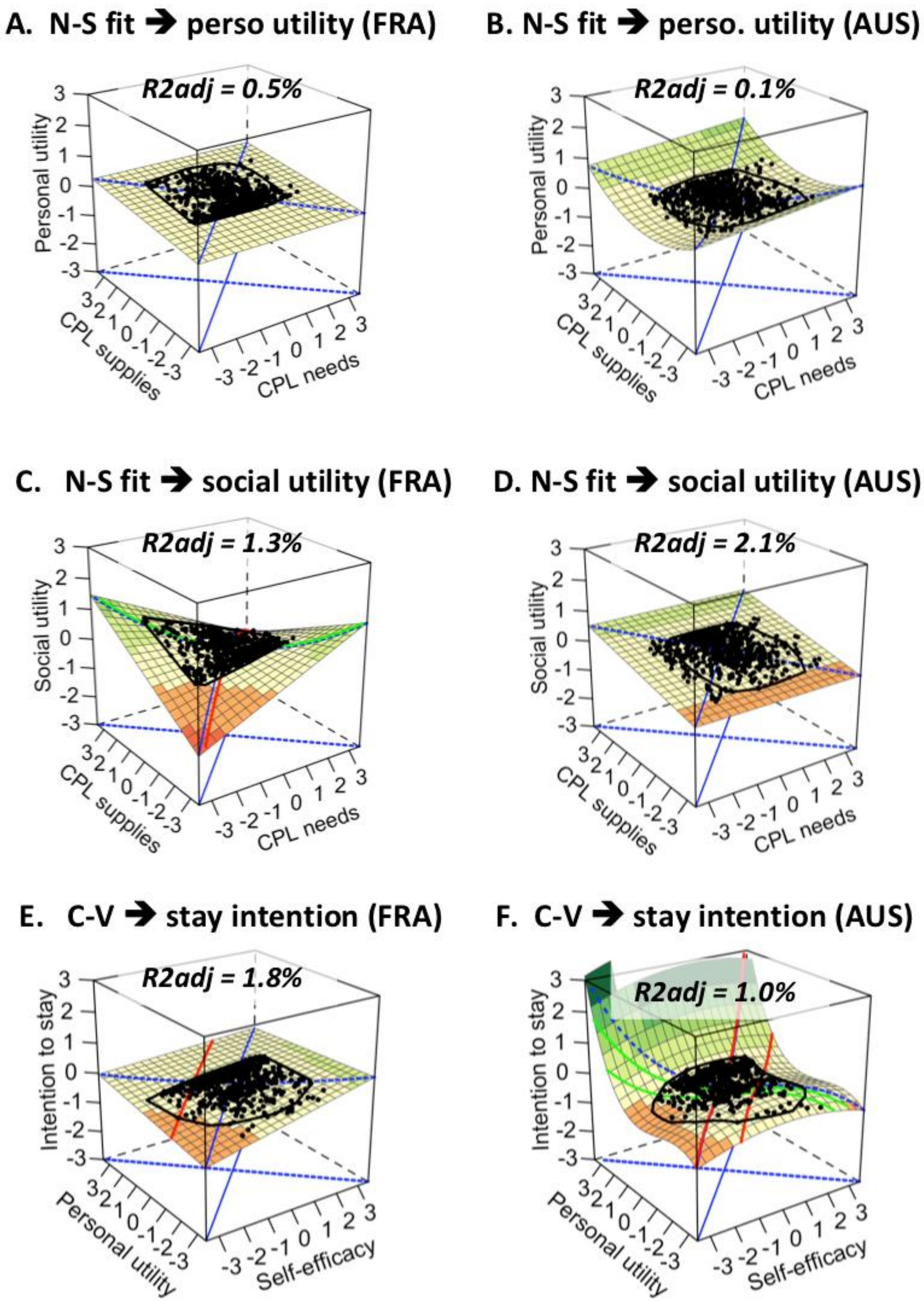

Note: Complementary response surfaces for negligible effects. CPL = Continuous professional learning; FRA= France; AUS = Australia. In both contexts, the combinatory effects of needs-supplies or control-value constructs is trivial, explaining 2% or less of the variance in the outcome, thus precluding meaningfull interpretation. Fit indices and model parameters are reported in Tables 3, 4 and 5 (main text).

Table S3. Polynomial models of CPL needs and supplies on teacher well-being

Variable	France			Australia		
	Enjoyment	Job satisfaction	Intention to stay	Enjoyment	Job satisfaction	Intention to stay
Parameters						
Intercept (b_0)	0.003	0.043	0.077	-0.036	0.000	0.000
Needs (b_1)	-0.134***	-0.065**	-0.181**	-0.130***	-0.053†	0.000
Supplies (b_2)	0.112***	-0.011	0.011	0.114***	0.205***	0.073**
Needs^2 (b_3)	0.000	0.021	-0.041	0.033*	0.000	0.000
Needs*Supplies (b_4)	0.000	-0.006	-0.048	-0.065*	0.000	0.000
Supplies^2 (b_5)	0.000	-0.097**	-0.039	0.000	0.000	0.000
Needs^3 (b_6)	0.000	0.000	0.019*	0.000	0.000	0.000
Needs^2*Supplies (b_7)	0.000	0.060***	0.056*	0.000	0.000	0.000
Needs*Supplies^2 (b_8)	0.000	0.000	0.056*	0.000	0.000	0.000
Supplies^3 (b_9)	0.000	0.060***	0.019*	0.000	0.000	0.000
Metaparameters						
$u_1 = b_1 + b_2$	-0.022	-0.077	-0.171†	-0.016	0.153***	0.073**
$u_2 = b_3 + b_4 + b_5$	0.000	-0.082†	-0.127*	-0.033*	0.000	0.000
$u_3 = b_6 + b_7 + b_8 + b_9$	0.000	0.119***	0.149*	0.000	0.000	0.000
$v_1 = b_1 - b_2$	-0.247***	-0.054	-0.192***	-0.244***	-0.258***	-0.073**
$v_2 = b_3 - b_4 + b_5$	0.000	-0.070	-0.032	0.098*	0.000	0.000
$v_3 = b_6 - b_7 + b_8 - b_9$	0.000	-0.119***	0.000	0.000	0.000	0.000
Polynomial family	FM3	FM25	FM21	FM9	FM3	FM2
wAIC	.127	.346	.128	.062	.125	.291
R2adj	.028	.024	.022	.035	.045	.005
CFI	1.000	1.000	1.000	1.000	0.992	1.000
RMSEA	0.000	0.000	0.000	0.000	0.007	0.000
SRMR	0.009	0.002	0.002	0.008	0.006	0.007

Note. Metaparameters u_1 to v_3 correspond to the linear, quadratic and cubic N-S fit (u_1 to u_3) or misfit (v_1 to v_3) effects. Zero values (0.000) are fixed by construction, as part of the identification strategy of the best-fitting polynomial model. Numbered models (e.g., “FM28”) correspond to the best-fitting polynomial family identified after comparison across 37 linear, quadratic, or cubic families. Response surfaces are reported in Fig. S4.

†p < .10; *p < .05; **p < .01; ***p < .001

Fig. S4 CPL needs-supplies fit effects on teacher well-being indicators

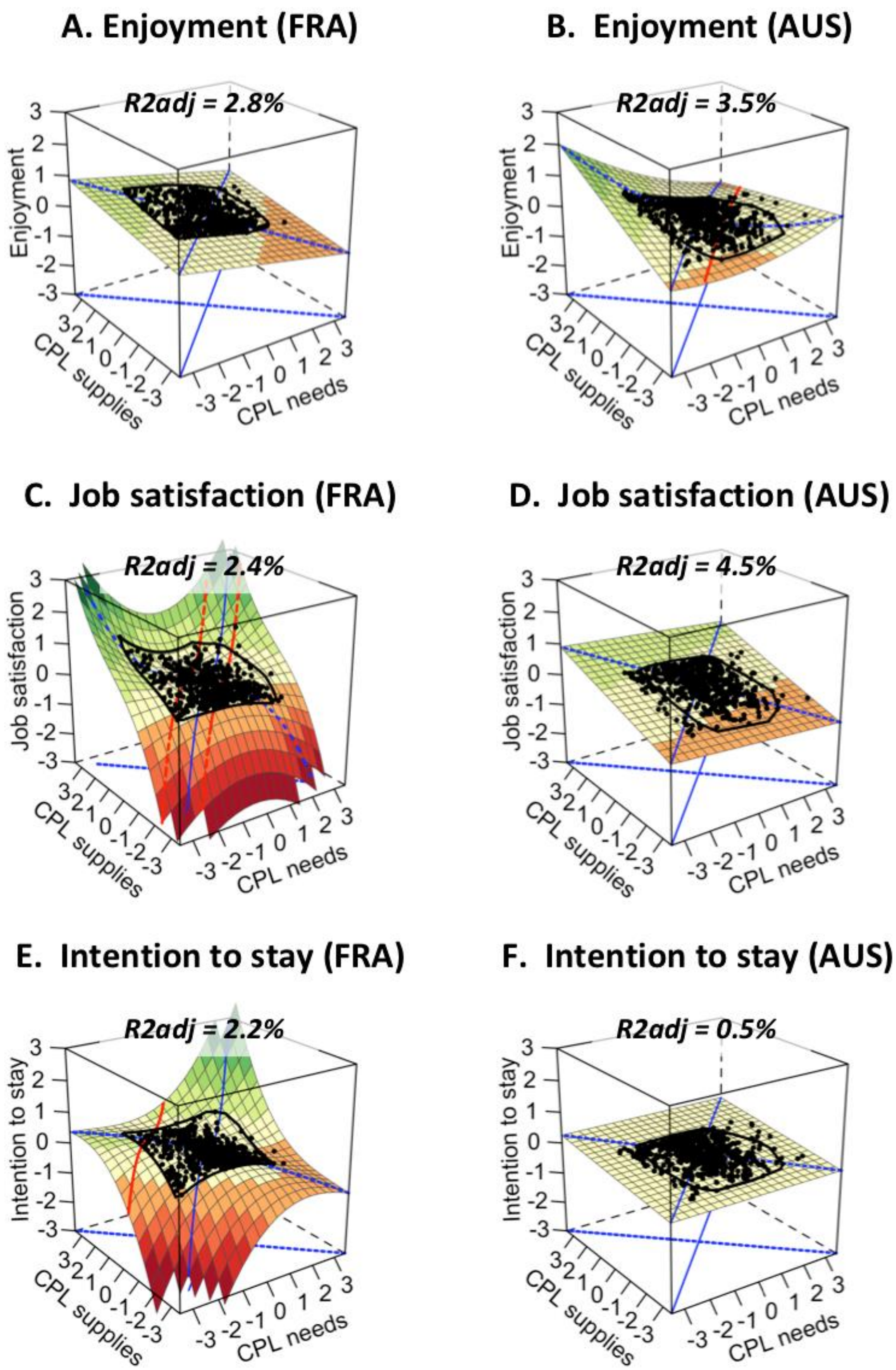

Note. CPL = Continuous professional learning; FRA= France; AUS = Australia. In both contexts, the combinatory effects of needs-supplies are nontrivial for, explaining more than 2% of the variance in the outcome, with a single exception (intention to stay in Australia). Models are reported in Table S3. Mediation analyses (section C) show that these N-S fit effects transit via self-efficacy, consistent with the integrative model of well-being (Fig. 1, main text).

D. Mediation Analyses

To further probe our integrative framework, we conducted mediation analyses examining whether the associations between CPL needs-supplies fit and teacher well-being indicators were transmitted via teacher self-efficacy. Due to a lack of available methodology for mediation process involving dual cubic models (i.e., one for N-S fit effects, one for C-V effects), we explored simplified models in which the mediator (self-efficacy) is linearly related to the outcome. To this aim, we relied on the mediation approach for quadratic polynomials proposed by Edwards [18], extending it to cubic polynomials to match our analyses.

Results in both French (Table S4) and Australian (Table S5) samples showed that all key polynomial effects, notably higher-order effects defining the polynomial family of each solution, showed significant indirect effects via self-efficacy. Including self-efficacy as a mediator also resulted in reducing the significance and size of N-S fit effects on well-being indicators, consistent with a mediation process. Although simplified by construction, these mediation models explained a substantial proportion of variance in enjoyment and job satisfaction (between 7% and 13%), and a nontrivial amount in stay intention (around 3%). These results highlight the importance of the linkage between N-S fit and C-V processes in relation to teacher well-being. More generally, these results confirm that N-S fit effects transit through the complex of C-V interactions via self-efficacy variations, thereby corroborating the integrative framework of this study (Fig. 1, main text).

Table S4. Mediation of N-S fit effects on teacher well-being by self-efficacy (France)

	Enjoyment	Job satisfaction	Intention to stay
Outcome: Self-efficacy			
Needs (b_1)	-0.194 (-0.253,-0.136)	-0.194 (-0.252,-0.135)	-0.194 (-0.253,-0.136)
Supplies (b_2)	0.031 (-0.028,0.09)	0.033 (-0.026,0.092)	0.032 (-0.027,0.091)
Needs^2 (b_3)	0.027 (-0.012,0.066)	0.027 (-0.012,0.066)	0.027 (-0.012,0.066)
Needs*Supplies (b_4)	0.005 (-0.047,0.056)	0.004 (-0.047,0.056)	0.005 (-0.047,0.056)
Supplies^2 (b_5)	0.031 (-0.014,0.077)	0.031 (-0.015,0.077)	0.032 (-0.014,0.077)
Needs^3 (b_6)	-0.021 (-0.032,-0.009)	-0.021 (-0.032,-0.009)	-0.02 (-0.032,-0.009)
Needs^2*Supplies (b_7)	0.062 (0.027,0.096)	0.062 (0.027,0.097)	0.061 (0.027,0.096)
R2 (% explained variance)	8.1	8.1	8.1
Outcome: Well-being indicator			
Self-efficacy (m_1)	0.349 (0.3,0.398)	0.283 (0.242,0.325)	0.089 (0.037,0.140)
Needs (b'_1)	-0.032† (-0.098,0.033)	0.009 (-0.038,0.057)	-0.149 (-0.262, -0.036)
Supplies (b'_2)	0.059† (0.01,0.109)	-0.018 (-0.101,0.065)	0.005 (-0.088, 0.097)
Needs^2 (b'_3)	0 (fixed)	0.013 (-0.025,0.050)	-0.043 (-0.106, 0.019)
Needs*Supplies (b'_4)	0 (fixed)	-0.008 (-0.056,0.041)	-0.041 (-0.127 ,0.045)
Supplies^2 (b'_5)	0 (fixed)	-0.095 (-0.156,-0.035)	-0.043 (-0.112, 0.026)
Needs^3 (b'_6)	0 (fixed)	0 (fixed)	0.018† (0.001, 0.034)
Needs^2*Supplies (b'_7)	0 (fixed)	0.051† (0.023,0.080)	0.053† (0.004, 0.102)
Needs*Supplies^2 (b'_8)	0 (fixed)	0 (fixed)	0.053† (0.004, 0.102)
Supplies^3 (b'_9)	0 (fixed)	0.051† (0.023,0.08)	0.018† (0.001, 0.034)
R2 (% explained variance)	13.6	10.1	3.0
Indirect N-S fit effects (mediation by self-efficacy)			
Needs ($b_1 * m_1$)	-0.068* (-0.090, -0.046)	-0.055* (-0.073, -0.037)	-0.017* (-0.029, -0.006)
Supplies ($b_2 * m_1$)	0.011 (-0.010, 0.032)	0.009 (-0.007, 0.026)	0.003 (-0.003, 0.008)
Needs^2 ($b_3 * m_1$)	0.009 (-0.004, 0.023)	0.008 (-0.003, 0.019)	0.002 (-0.001, 0.006)
Needs*Supplies ($b_4 * m_1$)	0.002 (-0.016, 0.020)	0.001 (-0.013, 0.016)	0.000 (-0.004, 0.005)
Supplies^2 ($b_5 * m_1$)	0.011 (-0.005, 0.027)	0.009 (-0.004, 0.022)	0.003 (-0.002, 0.007)
Needs^3 ($b_6 * m_1$)	-0.007* (-0.011, -0.003)	-0.006* (-0.009, -0.002)	-0.002* (-0.003, -0.001)
Needs^2*Supplies ($b_7 * m_1$)	0.021* (0.009, 0.034)	0.018* (0.007, 0.028)	0.005* (0.001, 0.010)

Note. Estimated effects and their 95% confidence intervals are reported. Indirect effects are computed as the product of the target polynomial effect on self-efficacy (e.g., b_1) and the effect of self-efficacy on the outcome. Effects are specified according to the best-fitting polynomial for self-efficacy, including equality and zero constraints (see Table 4, main text).

†Key N-S fit effects (family parameters) reducing in significance or size after including self-efficacy as a mediator.

*Significant mediation effects by self-efficacy (95% CI did not contain zero).

Table S5. Mediation of N-S fit effects on teacher well-being by self-efficacy (Australia)

	Enjoyment	Job satisfaction	Intention to stay
Outcome: Self-efficacy			
Needs (b_1)	-0.240 (-0.285,-0.195)	-0.241 (-0.286,-0.196)	-0.241 (-0.286,-0.196)
Supplies (b_2)	0.220 (0.182,0.258)	0.220 (0.181,0.258)	0.220 (0.182,0.258)
Needs^2 (b_3)	0.102 (0.07,0.133)	0.102 (0.07,0.134)	0.102 (0.07,0.134)
R2 (% explained variance)	12.4	12.4	12.4
Outcome: Well-being indicator			
Self-efficacy (m_1)	0.323 (0.271,0.375)	0.180 (0.135,0.224)	0.043 (-0.008,0.095)
Needs (b'_1)	-0.048 (-0.119,0.023)	-0.010† (-0.066,0.045)	0 (fixed)
Supplies (b'_2)	0.040 (-0.014,0.093)	0.165† (0.124,0.206)	0.062† (0.010,0.115)
Needs^2 (b'_3)	0.021† (-0.004,0.047)	0 (fixed)	0 (fixed)
Needs*Supplies (b'_4)	-0.042† (-0.094,0.009)	0 (fixed)	0 (fixed)
R2 (% explained variance)	12.6	7.4	0.1
Indirect N-S fit effects (mediation by self-efficacy)			
Needs ($b_1 * m_1$)	-0.078* (-0.096,-0.059)	-0.043* (-0.057,-0.03)	0.003 (-0.002,0.009)
Supplies ($b_2 * m_1$)	0.071* (0.054,0.088)	0.039* (0.028,0.051)	0 (fixed)
Needs^2 ($b_3 * m_1$)	0.033* (0.021,0.044)	0.018* (0.011,0.025)	0 (fixed)

Note. Estimated effects and their 95% confidence intervals are reported. Indirect effects are computed as the product of the target polynomial effect on self-efficacy (e.g., b_1) and the effect of self-efficacy on the outcome. Effects are specified according to the best-fitting polynomial for self-efficacy, including equality and zero constraints (see Table 4, main text).

†Key N-S fit effects (family parameters) reducing in significance or size after including self-efficacy as a mediator.

*Significant mediation effects by self-efficacy (95% CI did not contain zero).

References in Supporting Information

1. Chen, F. F. Sensitivity of goodness of fit indexes to lack of measurement invariance. *Struct. Equ. Model. Multidiscip. J.* **14**, 464–504 (2007).
2. Hu, L. & Bentler, P. M. Fit indices in covariance structure modeling: Sensitivity to underparameterized model misspecification. *Psychol. Methods* **3**, 424–453 (1998).
3. Jorgensen, T. D., Kite, B. A., Chen, P.-Y. & Short, S. D. Permutation randomization methods for testing measurement equivalence and detecting differential item functioning in multiple-group confirmatory factor analysis. *Psychol. Methods* **23**, 708–728 (2018).
4. Núñez-Regueiro, F. *et al.* Optimal Margins in Demands–Resources Fit and Student Engagement. (2025).
5. Núñez-Regueiro, F., Juhel, J. & Wang, M.-T. Does need satisfaction reflect positive needs-supplies fit or misfit? A new look at autonomy-supportive contexts using cubic response surface analysis. *Soc. Psychol. Educ.* **28**, 43 (2025).
6. Núñez-Regueiro, F. & Juhel, J. Model-Building Strategies in Response Surface Analysis. Preprint at https://hal.science/hal-05241391v1 (2022).
7. Núñez-Regueiro, F. & Juhel, J. RSAtools: Advanced response surface analysis. (2024).
8. Humberg, S., Schönbrodt, F. D., Back, M. D. & Nestler, S. Cubic response surface analysis: Investigating asymmetric and level-dependent congruence effects with third-order polynomial models. *Psychol. Methods* **27**, 622–649 (2022).
9. Schönbrodt, F. D. Testing fit patterns with polynomial regression models. Preprint at https://doi.org/10.31219/osf.io/ndggf (2016).
10. Kline, R. B. *Principles and Practice of Structural Equation Modeling*. (Guilford Publications, 2023).
11. *Model Selection and Multimodel Inference: A Practical Information-Theoretic Approach*. (Springer New York, New York, NY, 2002). doi:10.1007/b97636.
12. Wagenmakers, E.-J. & Farrell, S. AIC model selection using Akaike weights. *Psychon. Bull. Rev.* **11**, 192–196 (2004).
13. Cavenaugh, J. S. Bootstrap cross-validation improves model selection in pharmacometrics. *Stat. Biopharm. Res.* **14**, 168–203 (2022).

14. Hu, L. & Bentler, P. M. Cutoff criteria for fit indexes in covariance structure analysis: Conventional criteria versus new alternatives. *Struct. Equ. Model. Multidiscip. J.* **6**, 1–55 (1999).

15. Preacher, K. J. & Yaremych, H. E. Model selection in structural equation modeling. in *Handbook of Structural Equation Modeling* (ed. Hoyle, R. H.) (Guilford Publications, 2022).

16. Cohen, J. *Statistical Power Analysis for the Behavioral Sciences*. (Lawrence Earlbaum Associates, 1988).

17. Lenhard, W. & Lenhard, A. Computation of effect sizes. *Psychometrica* https://www.psychometrica.de/effect_size.html (2022) doi:10.13140/RG.2.2.17823.92329.

18. Edwards, J. R. Mediated Polynomial Regression. https://tarheels.live/jeffreyedwardswebsite/home/resources/mediated-polynomial-regression/.
